# Rice XB15, a Protein Phosphatase 2C, Negatively Regulates Cell Death and XA21-Mediated Innate Immunity

**DOI:** 10.1371/journal.pbio.0060231

**Published:** 2008-09-23

**Authors:** Chang-Jin Park, Ying Peng, Xuewei Chen, Christopher Dardick, DeLing Ruan, Rebecca Bart, Patrick E Canlas, Pamela C Ronald

**Affiliations:** 1 Department of Plant Pathology, University of California Davis, Davis, California, United States of America; 2 Agricultural Research Service, Appalachian Fruit Research Station, United States Department of Agriculture, Kearneysville, West Virginia, United States of America; University of North Carolina at Chapel Hill, United States of America

## Abstract

Perception of extracellular signals by cell surface receptors is of central importance to eukaryotic development and immunity. Kinases that are associated with the receptors or are part of the receptors themselves modulate signaling through phosphorylation events. The rice (Oryza sativa L.) XA21 receptor kinase is a key recognition and signaling determinant in the innate immune response. A yeast two-hybrid screen using the intracellular portion of XA21, including the juxtamembrane (JM) and kinase domain as bait, identified a protein phosphatase 2C (PP2C), called XA21 binding protein 15 (XB15). The interaction of XA21 and XB15 was confirmed in vitro and in vivo by glutathione-S-transferase (GST) pull-down and co-immunoprecipitation assays, respectively. XB15 fusion proteins purified from Escherichia coli and from transgenic rice carry PP2C activity. Autophosphorylated XA21 can be dephosphorylated by XB15 in a temporal- and dosage-dependent manner. A serine residue in the XA21 JM domain is required for XB15 binding. *Xb15* mutants display a severe cell death phenotype, induction of pathogenesis-related genes, and enhanced XA21-mediated resistance. Overexpression of *Xb15* in an XA21 rice line compromises resistance to the bacterial pathogen Xanthomonas oryzae pv. *oryzae*. These results demonstrate that *Xb15* encodes a PP2C that negatively regulates the XA21-mediated innate immune response.

## Author Summary

Resistance to pathogens is critical to plant and animal survival. Plants, unlike animals, lack an adaptive immune system and instead rely on the innate immune response to protect against infection. To elucidate the molecular mechanism of plant innate immunity, we are studying the signaling cascade mediated by the rice pathogen recognition receptor kinase XA21, which confers resistance to the bacterial pathogen *Xanthomonas oryzae* pv. *oryzae*. We demonstrate that XA21 binding protein 15 (a protein phosphatase 2C) negatively regulates XA21-mediated signaling resistance. This finding provides significant insight into regulation of receptor kinase-mediated immunity.

## Abstract 3

The rice protein phosphatase 2C XB15 is a negative regulator of pathogen recognition receptor kinase-mediated cell death and innate immunity.

## Introduction

Protein kinases regulate most cellular signal transduction pathways including cell growth and proliferation, cellular differentiation, morphogenesis, gene transcription, and immunity [1–3]. Adaptive immunity, restricted to vertebrates, is characterized by the creation of antigen-specific receptors through somatic recombination in maturing lymphocytes [4]. In contrast, innate immunity, common to both animals and plants, is mediated by a set of defined receptors referred to as pathogen recognition receptors (PRRs). Recognition of pathogen-associated molecular patterns (also called microbe-associated molecular patterns) or pathogen-derived avirulence (Avr) molecules by PRRs triggers signal transduction pathways mediated by activation of mitogen-associated protein kinase (MAPK) cascades and transcription factors [4,5]. These pathways lead to a core set of defense responses including accumulation of defense related molecules, increases in reactive oxygen species, calcium fluxes, and programmed cell death (PCD) [4–6].

In animals, recognition of pathogen-associated molecular patterns in extracellular compartments or at the cell surface is largely carried out by the Toll-like receptor (TLR) family containing leucine rich repeats (LRRs) in the extracellular domain [6]. TLRs associate with the interleukin-1 receptor-associated kinase (IRAK) family [7] and with receptor interacting-protein (RIP) kinases [8] via adaptor proteins. In plants, cell surface recognition of pathogen-associated molecular patterns or pathogen-derived Avr molecules is largely carried out by the non-RD class of receptor kinases (RKs) [6,9].

These “non-RD” kinases typically carry a cysteine (C) or glycine (G) before the conserved aspartate (D) residue. In contrast, the larger group of “RD” kinases have an arginine (R) immediately preceding the conserved catalytic aspartate (D) [10,11]. The RD class of kinases includes nearly all receptor tyrosine kinases and most characterized plant receptor serine/threonine kinases [10]. The non-RD class includes members of human IRAKs and RIPs, Drosophila Pelle, and members of plant RKs belonging to the IRAK family [10,12,13].

Plant genome analyses have revealed the presence of a large family of these non-RD IRAK kinases, with more than 45 encoded in the *Arabidopsis* genome and more than 370 found in the rice genome [10,14]. Members include the *Arabidopsis* flagellin RK (FLS2), the *Arabidopsis* elongation factor Tu RK (EFR) [15,16], the rice XA26 and Pi-d2 RKs [17–19], and the rice XA21 RK that mediates recognition of the Gram negative bacteria Xanthomonas oryzae pv. *oryzae* (*Xoo*) [18,20]. Because the presence of the non-RD motif in IRAK kinases is correlated with a role of the protein in pathogen recognition, there is great interest in understanding how they are regulated [10,21]. Unlike the majority of RD kinases, where phosphorylation of the activation loop is critical for activation [22], the mechanism of non-RD kinase regulation, in which many non-RD kinases do not autophosphorylate the activation loop, remains to be elucidated [10]. The juxtamembrane (JM) domain, a region of the RK that is N terminal to the kinase domain, has been suggested to be important for regulation of non-RD RKs and to serve as a high affinity binding site for downstream signaling proteins [10,23].

Although non-RD IRAK kinases are clearly essential for innate immunity in both plants and animals, sustained or highly induced immune response can be harmful [24]. It is therefore necessary that PRR signaling through non-RD kinases be under tight negative regulation. For example, misregulation of the non-RD IRAK1 in mice induces activation of nuclear factor κB (NF-κB) and increases inflammatory responses to bacterial infection [25,26].

Dephosphorylation of kinases by protein phosphatases (PPs) is a common mechanism for downregulating kinase-mediated signaling [27]. PPs are classified into two major classes: tyrosine phosphatases and serine/threonine phosphatases, depending on their substrates [27,28]. In humans, a group of MAPK phosphatases (MKPs) including MKP1, MKP5, and dual specificity PPs, negatively regulate the innate immune response [24,27,29].

In contrast to animal systems, negative regulation of kinases involved in plant innate immunity is not well understood. One important class of negative regulators are PP 2Cs (PP2Cs), a group of serine/threonine phosphatases that function as monomers and require Mn^2+^ and/or Mg^2+^ for activity [28,30]. One of the best characterized plant PP2Cs is the *Arabidopsis* kinase-associated PP (KAPP), which interacts with many RKs including FLS2, CLAVATA1 (CLV1), somatic embryogenesis RK 1, brassinosteroid-insensitive 1 (BRI1), and BRI1-associated RK 1 [31–36]. Overexpression of KAPP in *Arabidopsis* results in loss of sensitivity to flagellin treatment, suggesting that KAPP negatively regulates the FLS2-mediated defense response [33]. *Arabidopsis* CLV1 controls stem cell identity in shoot and flower meristems. In vitro, a CLV1 fusion protein can phosphorylate KAPP. Conversely, KAPP dephosphorylates the kinase domain of CLV1 in vitro [3,31,37,38]. So far, KAPP is the only PP known to be involved in regulation of RK-mediated signaling and to be associated with RKs in plants [31,33].

The rice XA21 RK is one of a few plant PRRs that has been studied in depth [18]. Despite the clear biological role for XA21 in the rice innate immune response, very little is known about the mechanism by which XA21-mediated resistance is regulated. Although the rice KAPP protein emerged as a good candidate for being a negative regulator of the XA21-mediated innate immune response, it does not interact with XA21 [39]. This suggests the presence of another protein that negatively regulates XA21-mediated signaling pathway.

In this study, we report the identification and characterization of rice XA21 binding protein 15 (XB15), which encodes a novel PP2C. Transgenic rice lines overexpressing *Xb15* display compromised *Xa21*-mediated resistance to *Xoo* strains carrying AvrXa21 activity. Conversely, *Xb15 Tos17* insertion mutants display cell death, constitutive induction of PR genes, and enhanced XA21-mediated resistance upon *Xoo* infection. Subsequent biochemical experiments show that XB15 can dephosphorylate autophosphorylated XA21 in a temporal- and dosage-dependent manner and that a serine residue in the XA21 JM domain is required for XB15 binding. Our findings are consistent with a model in which XB15 associates with the XA21 JM domain to negatively regulate the XA21-mediated innate immune response and cell death.

## Results

### XB15 Is an XA21 Binding Protein

The rice XA21 protein is representative of the large class of non-RD RKs predicted to be involved in plant innate immunity [10,18,20]. To elucidate the mechanism of XA21-mediated resistance and to identify its interaction partners, we performed a GAL4-based yeast two-hybrid screen using a rice cDNA library constructed from Oryza sativa spp. Indica line, IRBB21. IRBB21 is an isogenic line of the Indica variety IR24 carrying an introgression at the *Xa21* locus [40]. It has been previously reported that both the JM region (the portion of the cytoplasmic domain between the transmembrane sequence and the kinase domain) and the C-terminal region of RKs, can serve as high affinity binding sites for downstream signaling proteins [23]. Therefore, the entire XA21 predicted intracellular region, including the JM, kinase, and C-terminal domains, called XA21K668 (668–1,025 amino acids), was used as bait for this screen. Of the 7 × 10^7^ transformants screened, a total of eight unique clones both grew on selective media lacking histidine and tested positive for β-galactosidase reporter gene activity. These clones were named XA21 binding proteins (XBs). We have previously reported characterization of another *Xb*, called *Xb10*, which encodes a WRKY transcription factor [41], and *Xb3*, which encodes an ubiquitin ligase [42]. No RKs were isolated in this screen.

**Figure 1:**
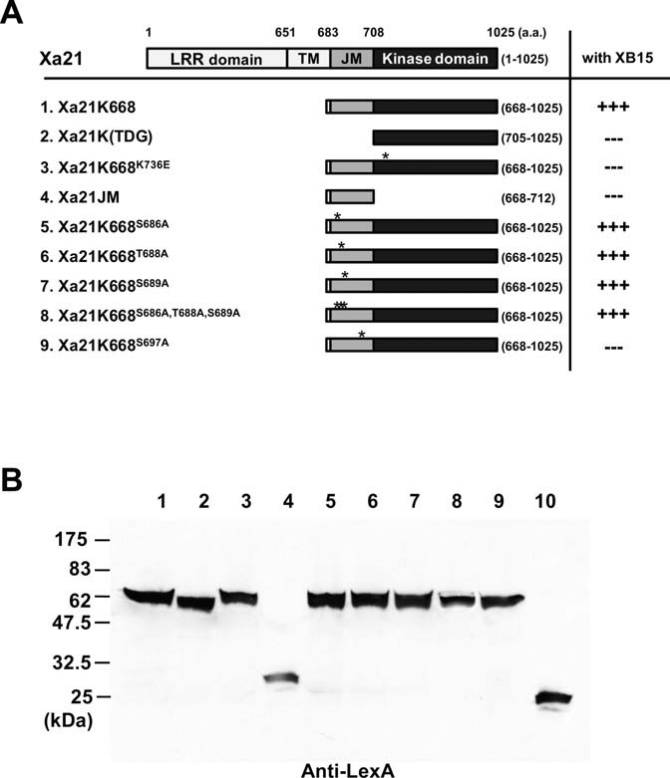
XB15 Interacts with XA21K668 in Yeast Two-Hybrid Assays (A) Schematic diagram of XA21 used as bait and the result of yeast two-hybrid analysis. Domains are as described in previous report [18]. Amino acid residue numbers and the total length of each XA21 constructs are indicated. (B) Protein gel blot analysis with protein extracted from yeast transformants. The extracts were prepared from the yeast strain pEGY48/p8op-LacZ cotransformed with (1) pAD-GAL4 containing full length of *Xb15* (AD-XB15) and pLexA containing XA21K668 (LexA-XA21K668), (2) AD-XB15 and LexA-XA21K(TDG), (3) AD-XB15 and LexA-XA21K668^K736E^, (4) AD-XB15 and LexA-XA21JM, (5) AD-XB15 and LexA-XA21K668^S686A^, (6) AD-XB15 and LexA-XA21K668^T688A^, (7) AD-XB15 and LexA-XA21K668^e^, (8) AD-XB15 and LexA-XA21K668^S686A,T688A,S689A^, (9) AD-XB15 and LexA-XA21K668^S697A^, or (10) AD-XB15 and pLexA that serves as a vector control. Using the with anti-LexA antibody, AD-XB15 (unpublished data) and LexA-XA21 mutants give bands corresponding to the expected mass of the fusion proteins. The vector control expresses a 24-kDa band corresponding to the LexA protein.

Here, we report the functional characterization of one of the isolated interacting proteins, XB15, the only PP among the isolated XBs. To assess whether the XA21 JM domain was important for the physical interaction of XB15 with XA21, we generated a new construct, XA21K(TDG) (residues 705–1,025), which lacks the JM domain. XA21K(TDG) was then used as bait in a yeast two-hybrid assay with XB15 (Figure 1A). Activation of the *lacZ* reporter gene was dependent on the simultaneous presence of pAD-XB15 and pLexA-XA21K668. XA21K(TDG) lacking the JM domain did not interact with XB15. This result suggests that the JM domain contains specific amino acids needed for XB15 binding in yeast and that these residues may serve as a docking site for XB15. The JM region (XA21JM) lacking the kinase domain was not sufficient for the interaction with XB15 (Figure 1A). XB15 also failed to interact with the catalytically inactive mutant XA21K668^K736E^, in which lysine 736 is substituted with glutamic acid [43], suggesting that XA21 kinase activity is also important for the interaction.

On the basis of these results, we hypothesized that a residue(s) in the JM domain phosphorylated by XA21 may serve as a docking site for XB15. It has previously been reported that XA21 autophosphorylates residues Ser686, Thr688, and Ser689 in vitro [44]. To test if these residues are important for XB15 binding, we assessed whether mutations in these sites affected binding activity. We found that all three XA21 single mutants, XA21^S688A^, XA21^T688A^, XA21^S689A^, as well as the triple mutant XA21^S688A,T688A,S689A^ maintained interaction with XB15. We next assessed the role of three previously uncharacterized Ser and Thr residues (Ser697, Ser699, and Thr705) in the JM region of XA21. When Ser697 is mutated to Alanine, interaction with XB15 is abolished (Figure 1A). This result indicates that Ser697 in the XA21 JM domain is critical for interaction with XB15.

To confirm that the absence of interaction was not due to lack of expression of the fusion constructs in yeast cells, we performed protein gel blot analysis with anti-XB15 or anti-LexA antibodies. All yeast cells transformed with *AD-Xb15* carried an 80-kDa band cross-reacting with the anti-XB15 antibody (unpublished data). In Western blot analysis using an Anti-LexA antibody, yeast cells expressing LexA-XA21K668 or -XA21 variants, displayed bands with molecular weights corresponding to the correct size of each fusion protein (Figure 1B). Yeast cells expressing the vector control produced a protein of 24 kDa that reacted with the anti-LexA antibody.

### *Xb15* Encodes a PP2C with Similarity to *Arabidopsis* POLTERGEIST

**Figure 2:**
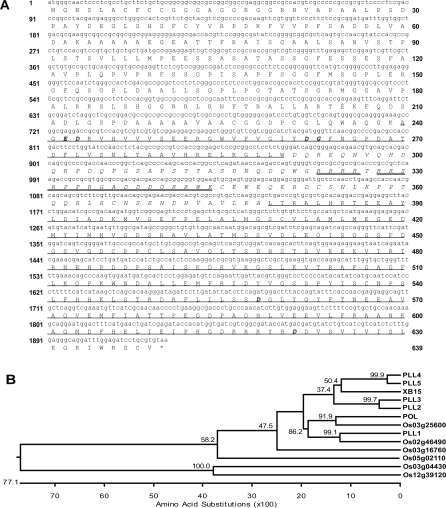
Phylogenetic Relationships among POL and PLL Genes from Rice and *Arabidopsis* (A) Deduced amino acid sequence of XB15. Italic bold represents residues that interact with metal cofactors in human PP2Cα [47]. The PP2C catalytic domain is underlined, and the unique insertion sequence in the PP2C catalytic domain is marked with italics. Predicted nuclear targeting signals are double-underlined (http://www.psort.org/).(B) Phylogenetic analysis at POL and PLLs from *Arabidopsis* and rice. For determining phylogenetic relationships, protein sequences of the conserved PP2C domains for putative plant proteins related to POL were aligned and then used in ClustalV. Ten thousand bootstrap replicates were performed. Rice PP2Cs with low homology to XB15, Os03g04430, and Os12g39120 were defined as outgroups. Sequences used in this analysis were as follows: POL, PLL1 (AAC3686), PLL2 (NP195860), PLL3 (NP187551), PLL4 (AAL38775), and PLL5 (AAK32783) from *Arabidopsis*; and XB15, Os03g60650, Os03g25600, Os02g46490, Os05g02110, and Os03g16760 from rice.

*Xb15* carries a 3,219-bp open reading frame that consists of three introns with lengths of 94 bp, 1,070 bp, and 138 bp and four exons. The ORF is predicted to encode a 639 amino acid protein (Figure 2A) with a molecular mass of 69.2 kDa and an isoelectric point of 5.4. XB15 is similar in overall structure to known plant PP2Cs, with a conserved C terminus (240–630 amino acids), which is predicted to have phosphatase catalytic activity (Figure 2A, underlines). It has a unique N terminus (1–239 amino acids) with no similarity to proteins with known function. The catalytic domain is interrupted by approximately 100 amino acids with no similarity to any sequences in the database (Figure 2A, with italics). XB15 contains six amino acids residues (four aspartic acids, one glutamic acid, and one glycine) known to interact with two Mg^2+^ or Mn^2+^ ions to form a binuclear metal center in *Arabidopsis* POL and human PP2Cα [45–47].

We next compared the PP2C domain of XB15 with other evolutionary related proteins from the reference plant species rice and *Arabidopsis*, such as *Arabidopsis* POL and POL-like proteins (PLLs) (Figure 2B). XB15 shows significant similarity to PP2Cs from other organisms including *Arabidopsis* POL (47% identity) and shares several features with the *Arabidopsis POL* and *PLL*s such as unique intron/exon boundaries and an insertion in the same region of the PP2C catalytic domain. Figure 2B shows the phylogenetic relationship among *POL* and *PLL* genes from rice and *Arabidopsis* based on the amino acid sequence of the last three exons. *Arabidopsis* POL and PLL1 cluster in one branch of the tree, consistent with the similar primary phenotype of *pol* and *pll1* mutants—phenotypic suppression of *clv* mutants [48,49]. These two proteins have also been shown to regulate the balance between stem-cell maintenance and differentiation and are closely related to *Wuschel,* which encodes a homeodomain transcription factor expressed in shoot meristems [50]. XB15 groups with *Arabidopsis* PLL2, PLL3, PLL4, and PLL5, showing the greatest similarity to PLL4 and PLL5 with 57.5% and 56.3% identity, respectively. *pll4* and *pll5* mutants develop abnormal leaves that are altered in shape [48]. To date, no function has been reported for *Arabidopsis* PLL2 or PLL3, or any of the rice PLLs including XB15, leaving the functional role of these proteins unknown.

### XB15 Functions as a Negative Regulator in the XA21-Mediated Innate Immune Response

**Figure 3:**
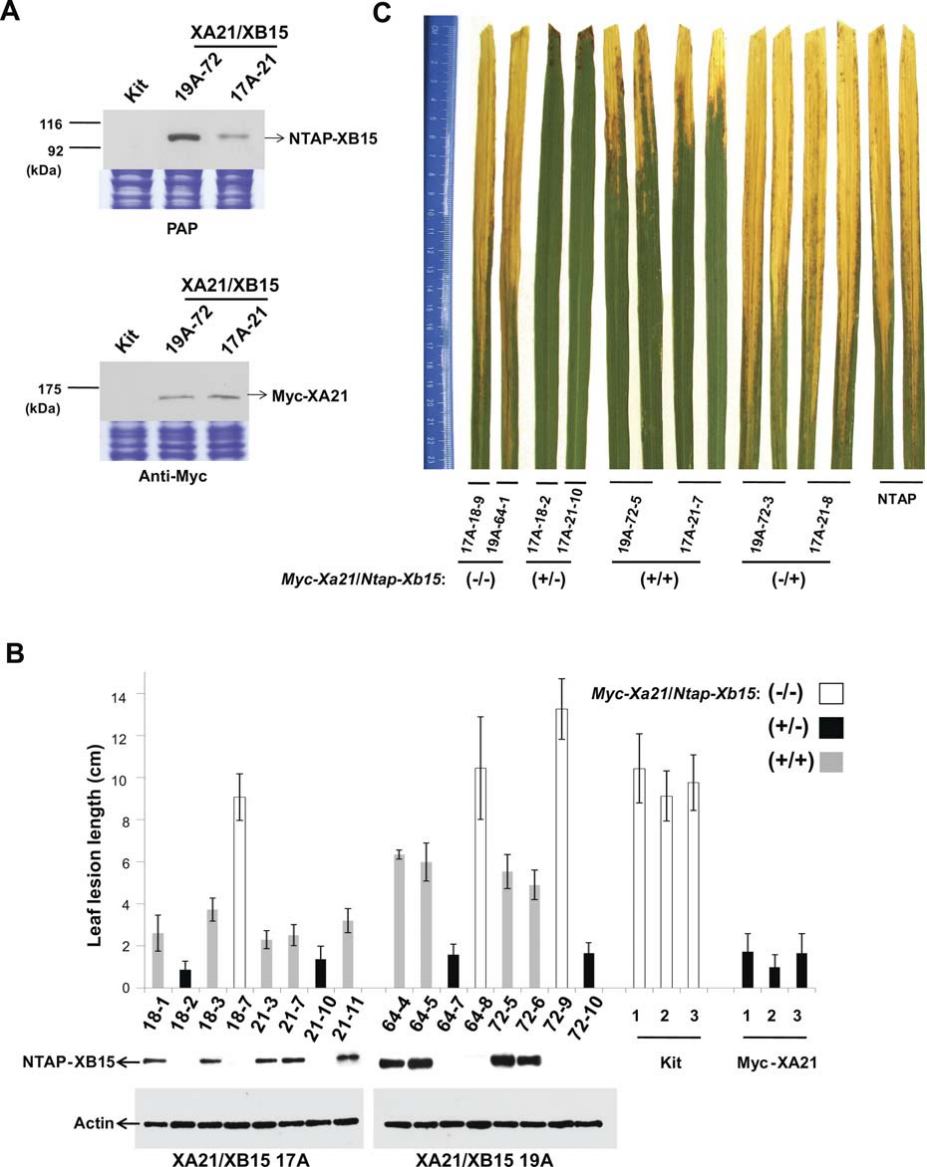
XA21 Rice Plants Overexpressing *Xb15* Are Susceptible to *Xoo* (A) Immunodetection of Myc-XA21 and NTAP-XB15. Equal amounts (50 μg) of total protein form Kitaake wild type (Kit) and progeny (F_1_) from a cross with Myc-XA21 and NTAP-XB15 19A and 17A (19A-72 and 17A-21, respectively) extracted and analyzed by SDS-PAGE, and immunobloted with anti-Myc or PAP antibodies. Equal loading of total proteins was confirmed by Coomassie blue staining of protein (lower panels). (B) Phenotype/genotype cosegregation analysis of segregants (F_2_) of XA21/XB15 19A and 17A. Segregants of 19A-64, 19A-72, 17A-18, and 17A-21, Kitaake wild type (Kit), and transgenic rice carrying *Myc-Xa21* (Myc-XA21) were inoculated at 6-wk-old age, and the lesion lengths were measured 12 d after inoculation. Each data point represents the average and standard deviation of at least four samples. Progeny carrying the *Xa21* alone or both of *Xa21* and *Xb15* are labeled with a black bar (+/−) or gray bar (+/+). Progeny missing both genes are labeled with a white bar (−/−). Immunodetection of XB15 in the XA21/XB15 17A and 19A is shown under the graph for each segregant. Total protein was extracted from each plant and protein gel blot analysis was performed with PAP antibody and anti-actin antibody to detect NTAP-XB15 and actin protein, respectively. (C) Water-soaked disease lesions on two leaves from four genotype of segregants (F_2_) (*Myc-Xa21/Ntap-Xb15*; −/−, +/−, +/+, and −/+) and transgenic plants overexpressing NTAP only (NTAP) 16 d after *Xoo* PR6 inoculation.

To explore the role of Xb15 in resistance, we examined whether constitutive overexpression of *Xb15* would affect resistance to *Xoo*. We first generated an *Xb15* tagged N-terminal tandem affinity purification (NTAP) construct under the control of the ubiquitin promoter (*Ubi*) [51,52] and then introduced this construct into the rice cultivar Kitaake using our established transformation procedures [53,54]. The resulting transgenic plants (NTAP-XB15, pollen donor) were crossed with another Kitaake line possessing an N-terminal Myc-epitope-tagged XA21, under control of its native promoter (Myc-XA21, pollen recipient). The presence of the *Myc-Xa21* and/or *Ntap-Xb15* gene in the resulting F_1_ was confirmed by the PCR analysis (unpublished data). Next, we tested the expression of NTAP-XB15 and Myc-XA21 in the cross (XA21/XB15 17A-21 and 19A-72) using an anti-Myc antibody and peroxidase-anti-peroxidase (PAP, for detecting the TAP tag) (Figure 3A). Bands corresponding to the predicted molecular mass of Myc-XA21 and NTAP-XB15 were detected at approximately 95 and 140 kDa, respectively confirming that the crossing was successful and that the progeny express both NTAP-XB15 and Myc-XA21. Although equivalent amounts of protein were analyzed, no signals were observed in nontransgenic Kitaake plants.

Six-week-old progeny from self-pollinated XA21/XB15 17A-18, 17A-21, 19A-64, and 19A-72 were then inoculated with *Xoo* PR6 strain PXO99 expressing AvrXA21 and examined for cosegregation of genotype with phenotype by PCR analysis and measurement of the length of *Xoo*-induced lesions. All *Myc-Xa21* plants overexpressing *Ntap-Xb15* (*Myc-Xa21*/*Ntap-Xb15*, +/+) by PCR analysis showed accumulation of NTAP-XB15 by protein gel blot analysis and displayed enhanced susceptibility to *Xoo* PR6 compared to transgenic plants carrying *Myc-Xa21* alone (+/−) (Figure 3B). Western blot analysis revealed a higher accumulation of NTAP-XB15 protein in XA21/XB15 19A line compared to XA21/XB15 17A, which correlated with longer leaf lesions developed by *Xoo* PR6, indicating that XB15 negatively regulates the XA21-mediated defense pathway in a dosage-dependent manner.

We could barely distinguish a difference in XA21 accumulation with homozygous or heterozygous Myc-XA21 transgenic plants (Figure S1). Therefore, to rule out the possibility that this phenotypic difference is caused by a *Xa21* dosage effect, we selected segregants (F_2_) carrying *Myc-Xa21* and analyzed the next generation (F_3_) to determine which segregants were heterozygous or homozygous for *Myc-Xa21*. After PCR genotyping 20 progeny from each line, we identified 17A-18–1, 17A-18–2, and 19A-72–5 as homozygous for *Myc-Xa21* (unpublished data). The remaining segregants are heterozygous for *Myc-Xa21*. Inoculation analysis revealed that there is no correlation between the number of copies of *Myc-Xa21* and the enhanced susceptibility phenotype in the NTAP-XB15 overexpression lines (Figure 3B). These results indicate that the enhanced susceptibility observed in the crossing population (F_2_) is caused by NTAP-XB15 overexpression and is not due to the presence of fewer copies of *Myc-Xa21*.

Figure 3C shows a picture of two typical leaves from each of the following inoculated rice plants: F_2_ segregants of XA21/XB15 17A and 19A, and transgenic plants overexpressing NTAP only (NTAP) 16 d after *Xoo* PR6 inoculation. While the Myc-XA21 plants lacking *Ntap-Xb15* (+/−, XA21/XB15 17A-18–2 and 17A-21–10) were highly resistant, showing short lesions (approximately 2 cm), inoculated leaves of crossed lines (+/+), XA21/XB15 17A-21–7 and 19A-72–5, developed typical water-soaked, long lesions (approximately 8–10 cm). In inoculated NTAP-XB15 lines lacking *Xa21* (−/+, XA21/XB15 19A-72–3 and 17A-21–8), there was no significant difference from segregants not carrying *Myc-Xa21* and *Ntap-Xb15* (−/−) in lesion lengths. These results demonstrate that overexpression of XB15 reduces XA21-mediated resistance to *Xoo*.

**Figure 4:**
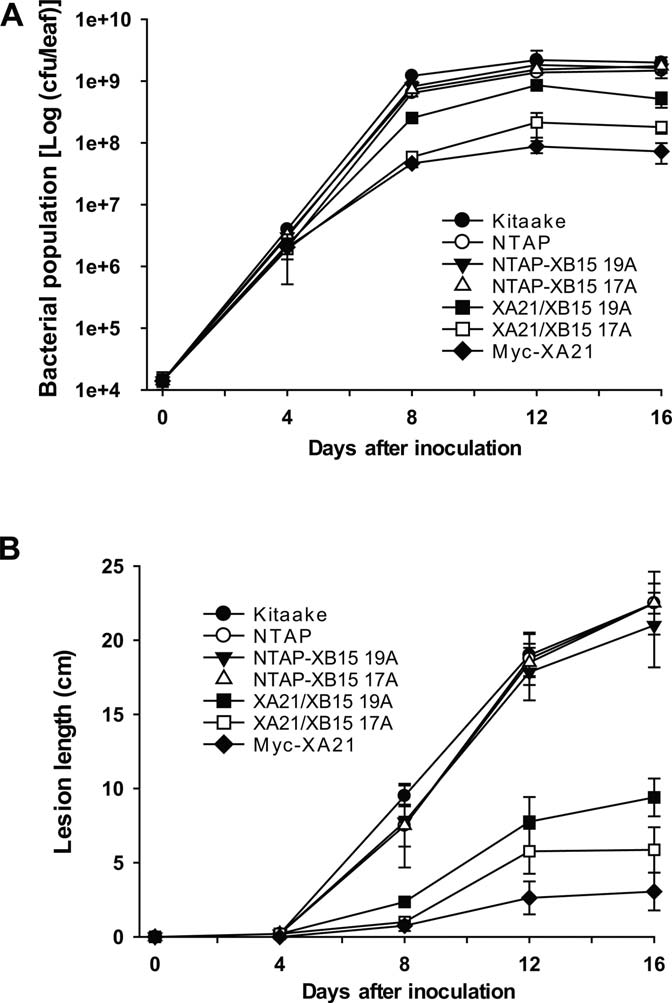
*Xoo* PR6 Growth Curves and Lesion Development in Rice Plants Overexpressing *Xb*15 (A) Plots of *Xoo* PR6 populations over 16 d in segregants (F_2_) of XA21/XB15 19A-72 and 17A-21, Kitaake, and NTAP lines. For each time point, the bacterial populations were separately determined for three leaves. Capped, vertical bars represent standard deviation of values (cfu/leaf) from three samples. Experiments were repeated over three times with similar results. NTAP-XB15 19A (*Myc-Xa21/Ntap-Xb15* [−/+], 19A-72–3, −7, and −12); NTAP-XB15 17A (−/+, 17A-21–4, −5, and −8); XA21/XB15 19A (+/+, 19A-72–5, −6, −8, and −11); XA21/XB15 17A (+/+, 17A-21–2, −3, −7, and 11); Myc-XA21 (+/−, 19A-72–1, −10 and 17A-21–6, −10). (B) Lesion length development of *Xoo* PR6 inoculated plants, segregants (F_2_) of XA21/XB15 19A-72 and 17A-21, Kitaake, and NTAP lines. Each data point represents the average and standard deviation of at least four samples. NTAP-XB15 19A (*Myc-Xa21/Ntap-Xb15* (−/+), 19A-72–12); NTAP-XB15 17A (−/+, 17A-21–8); XA21/XB15 19A (+/+, 19A-72–14); XA21/XB15 17A (+/+, 17A-21–15); Myc-XA21 (+/−, 17A-21–10).

We quantified the effect of XB15 overexpression on bacterial growth by monitoring bacterial populations on XA21/XB15 17A and 19A plants (F_2_) inoculated with *Xoo* PR6 (Figure 4A). For both growth curve and lesion length analysis up until 4 d after inoculation (DAI), there was no significant difference in bacterial growth in any of the lines. However, at 12 DAI, *Xoo* PR6 populations in the Kitaake and NTAP lines reached approximately 1.5 × 10^9^ colony-forming units per leaf (cfu/leaf): whereas, population in Myc-XA21 plants leveled off at fewer than 8.7 × 10^7^ cfu/leaf. In XA21/XB15 19A carrying *Myc-Xa21* and *Ntap-Xb15* (+/+, 19A-72–5, −6, −8, and −11), *Xoo* PR6 populations grew to 8.5 × 10^8^ cfu/leaf, a 10-fold increase compared to segregants carrying *Myc-Xa21* alone (+/−, 19A-72–1, −10 and 17A-21–6, −10). A significant difference in bacterial growth between the lines was observed continuously up to 16 DAI. We also measured the length of *Xoo*-induced lesions on all rice lines (Figure 4B). The progeny of XA21/XB15 19A and 17A (+/+, 19A-72–14 and 17A-21–15) displayed enhanced susceptibility to *Xoo* PR6 with lesions ranging in length from 4–10 cm compared to the segregants carrying *Myc-Xa21* alone (+/−, 17A-21–10), which showed 2–3 cm lesion lengths. Segregants overexpressing *Ntap-Xb15* but lacking *Myc-Xa21* (−/+) did not show significant difference in bacterial populations and lesion lengths, as compared to Kit and NTAP control.

### A Retrotransposon Insertional Line of *Xb15* Exhibits Cell Death

**Figure 5:**
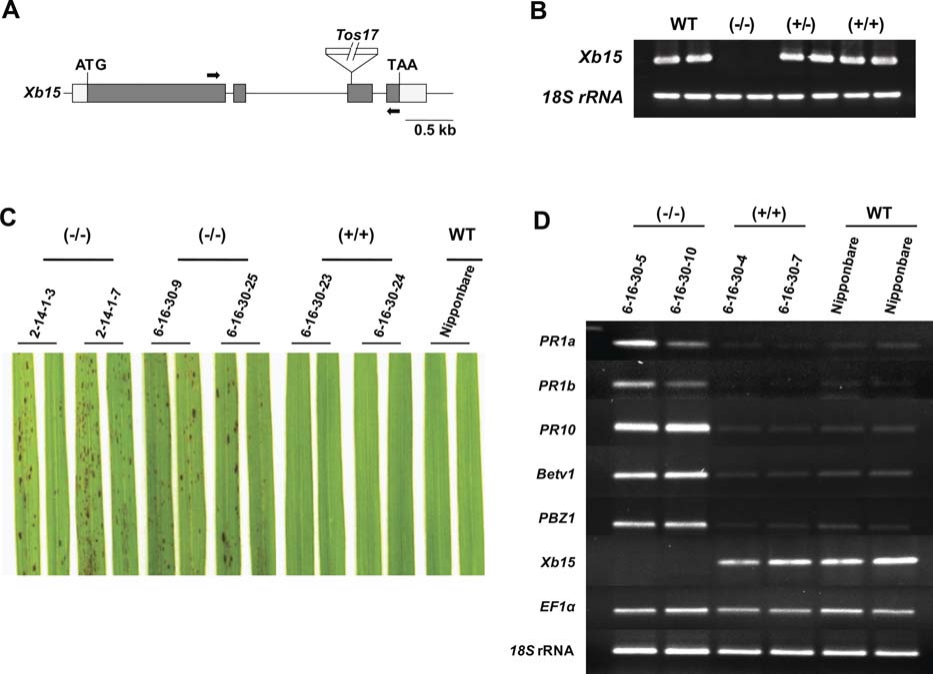
An *Xb15 Tos17* Mutant Exhibits Cell Death Lesions (A) Representation of *Xb15* genomic DNA structure and the position of the *Tos17* insertion site. Four exons (boxes) are separated by three introns (lines). The protein-coding regions are indicated by shaded boxes with the start codon (ATG) and stop codon (TAA). Gray boxes correspond to untranslated sequences. Arrows indicate locations of primers designed for RT-PCR. (B) The transcripts levels of *Xb15* in Nipponbare wild type (WT), homozygous *Tos17* insertion (−/−), heterozygote (−/+), no-insertion plants (+/+). Total RNA was extracted from fully extended 5-wk-old leaves. RT-PCR analysis was performed with Xb15 specific primers indicated in (A). 18S rRNA was used for an internal control. (C) Morphological phenotype of the *Tos17* insertional line (M_5_) of *Xb15*. Photographs were taken 6 wk after germination. Symbols for genotypes for *Tos17* in *Xb15* are as described above. (D) PR gene expressions in plants *Xb15 Tos17 mutants* (M_5_). Total RNA was extracted from 5-wk-old wild type and insertion mutant line plants, and RT-PCR was performed with specific primers for *PR1a*, *PR1b*, *PR10*, major birch allergen (*Betv1*), and *PBZ1*. Control RT-PCR reactions were carried out with *EF1α* and *18S* rRNA. Symbols for genotypes for *Tos17* in *Xb15* are same as described above.

To further investigate the in vivo function of *Xb15*, a knockout mutant line was identified from a collection of rice mutants generated by random insertion of the endogenous retrotransposon *Tos17* (http://tos.nias.affrc.go.jp/∼miyao/pub/tos17/) [55]. One mutant line (NF9014) carried a *Tos17* insertion in the third exon of the *Xb15* gene (Figure 5A). This insertion resulted in a lack of expression of the full-length *Xb15* mRNA compared to the wild type Nipponbare plants when tested with appropriate primers by reverse transcription-PCR (RT-PCR) (Figure 5B). 18S ribosomal RNA (18S rRNA) was used as an internal control. The homozygous insertion mutant line (−/−) showed a severe cell death phenotype marked by the appearance of small necrotic lesions that were apparent at the vegetative stage (Figure 5C). Of the 24 segregants of 6-16-30, six out of eight of the heterozygotes (+/−) displayed the cell death phenotype. Two of the heterozygotes displayed a severe cell death phenotype similar to that observed in the homozygous individuals (−/−). In contrast, all nine homozygous (−/−) plants displayed a cell death phenotype and seven of these were very severe (Figure S2).

We next tested if the cell death phenotype correlated with alterations in *PR* gene expression. Total RNA was extracted from the mutant line and the Nipponbare control. RT-PCR was performed with primers targeting PR genes as molecular markers. Figure 5D shows that the expression of full-length *Xb15* was not detected in the *Tos17* mutant line. In contrast, all the defense-related genes tested, *PR1a*, *PR1b*, *PR10*, *Betula verrucosa 1* (*Betv1*, major birch allergen), and probenazole-inducible protein 1 (*PBZ1*) were highly expressed in the insertion mutant lines (−/−) but barely expressed in Nipponbare wild type (WT) or the no-insertion *null* plants (+/+). Both the internal controls (*18S* rRNA and *elongation factor 1 α* [*EF1α*]) showed constitutive expressions in all tested plants.

To confirm that the cell death phenotype of the *Tos17* insertion mutant is due to the loss of function of XB15, we constructed a plasmid for the gene-specific knockdown of *Xb15* by RNA interference (RNAi) with an inverted repeat of a specific fragment of *Xb15* cDNA under control of the *Ubi* promoter. The protein level of XB15 in *Xb15* RNAi transgenic rice plants was severely reduced compared with the Kitaake control (Figure S3A). We observed a similar cell death to that detected in the *Tos17* insertion mutant line in several independent RNAi lines (Figure S3B). Taken together, these results show that loss-of-function of XB15 induces expression of defense-related genes and elicits a cell death phenotype, suggesting a negative regulatory role for *Xb15* in the defense signaling pathway.

### A Retrotransposon Insertional *Xb15* Mutant Carrying *Xa21* Shows Enhanced Resistance

We have shown that the *Xb15 Tos17* insertion mutant line (NF9014) accumulates PR gene transcripts (Figure 5). On the basis of this result, we hypothesized that these lines would be more resistant to *Xoo*. However, because Nipponbare plants are already moderately resistant to *Xoo* PR6 and because we are pushing the limits of sensitivity of our bacterial assay, we were unable to detect enhanced resistance in these lines (unpublished data).

To further explore the role of *Xb15* in resistance, we carried out additional experiments in the Kitaake genetic background, which is highly susceptible to *Xoo* PR6 (Figures 3 and 4). In this experiment, we crossed the *Xb15 Tos17* insertion mutant line (NF9014–1) (pollen recipient) with a Kitaake line containing Myc-XA21 under control of its native promoter (pollen donor). We then tested if the loss of XB15 function alters the XA21-mediated defense response. We were able to simultaneously test for both reduced and enhanced resistance by taking advantage of the fact that the resistance conferred by XA21 is developmentally regulated. At the juvenile two-leaf stage (∼2 wk old), the plants are fully susceptible. Full resistance develops only at the adult stage [56].

**Figure 6:**
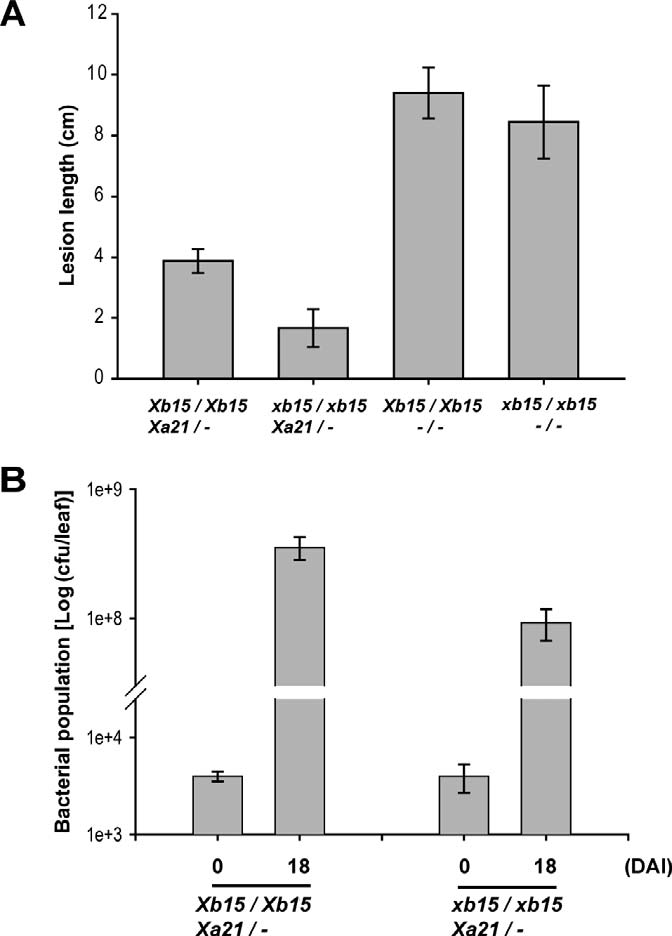
XA21 Rice Plants Carrying *Xb15 Tos17* Gene Exhibit Increased Resistance of *Xoo* (A) Three-week-old segregants (F_4_) of NF9014–1 (*Tos17 Xb15*) and Myc-XA21 were inoculated with *Xoo* PR6 and lesion lengths were measured 18 DAI. Each data point represents the average and standard deviation of at least three samples. (B) Bacterial populations were determined at 0 and 18 DAI. For each time point, the mean of the bacterial cfu/leaf from three plants per genotype is shown. Error bars indicate the standard deviation. The experiment was repeated three times with similar results.

We therefore inoculated progeny (F_4_) from the cross (NF9014–1/Myc-XA21) at 3 wk old when XA21 resistance is not yet fully active. At this stage of development, XA21 rice plants are only partially resistant (approximately 40% of resistance to that of 6-wk-old plants) [56]. Because lesion development continues to 16 d (Figure 4), we measured the lesion lengths at 18 d to capture as large a difference as possible (Figure 6A).

We found that progeny carrying *Xa21* and the *Xb15 Tos17* mutation (*xb15*/*xb15*, *Xa21*/−), displayed enhanced resistance to *Xoo* PR6 with lesions ranging length from 1–2 cm compared to the segregants carrying *Xa21* alone (*Xb15*/*Xb15*, *Xa21*/−), which showed approximately 4-cm lesion lengths. The difference was statistically significant. In contrast, although enhanced resistance was observed in the *Xb15 Tos17* mutant lines lacking *Xa21* (*xb15*/*xb15*, −/−) as compared to the wild-type *Xb15* line (*Xb15*/*Xb15*, −/−), this difference was not statistically significant (Figure 6A). This result indicates that, using our currently available methods, the enhanced resistance caused by the *Xb15* mutation can be detected only in the presence of XA21.

To further validate the enhanced resistance phenotype conferred by the *Xb15 Tos17* mutation in the XA21 genetic background, we measured bacterial populations in these lines. In Figure 6B, we show that *Xoo* populations multiplied to 9.2 × 10^7^ cfu/leaf in the XA21 plants carrying the *Xb15 Tos17* mutation (*xb15*/*xb15*, *Xa21*/−). In contrast, in the absence of the *Xb15 Tos17* mutation (*Xb15*/*Xb15*, *Xa21*/−), bacterial populations reached to 3.5 × 10^8^ cfu/leaf a 3.8-fold increase compared to the *Xb15 Tos17* mutant lines. These results clearly show that loss of XB15 function enhances XA21-mediated resistance.

### XB15 Has PP2C Activity

**Figure 7:**
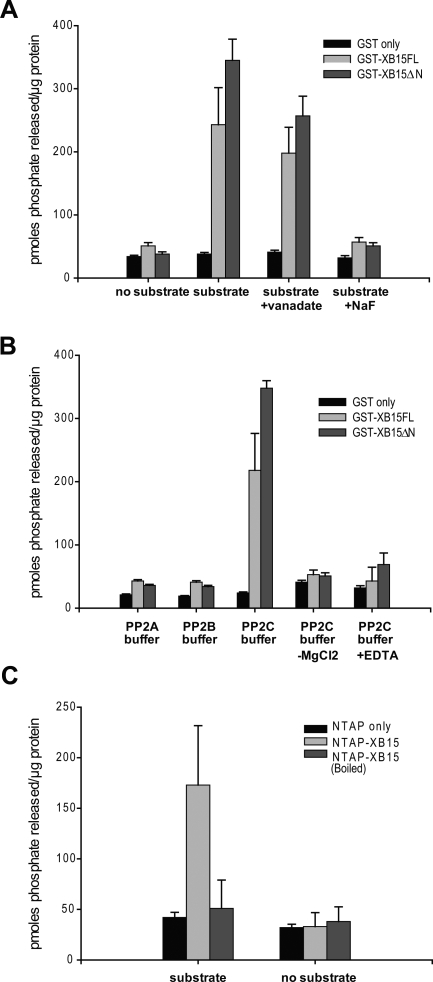
XB15 Possesses PP2C Catalytic Activity (A) XB15 protein is a serine/threonine phosphatase. Five micrograms of GST-XB15FL (full length XB15), GST-XB15ΔN (catalytic domain only), or GST was incubated with: no substrate, 100 μM substrate, 100 μM substrate plus 1 mM sodium vanadate, 100 μM substrate plus 50 mM NaF. (B) XB15 protein is a PP2C. Five micrograms of GST-XB15FL, GST-XB15ΔN, or GST was incubated with 100 μM substrate in each of the three buffers specific for PP2A, PP2B, and PP2C activity. (C) The XB15 protein purified from rice plants carries a PP2C activity. NTAP-XB15 and NTAP were purified from transgenic plants and 1 μg of each protein was incubated with 100 μM substrate in PP2C buffer. For the boiled NTAP-XB15, 1 μg of NTAP-XB15 was boiled in water for 20 min. The data are average value of three experiments. Error bars represent standard deviations.

To elucidate the mechanism of XB15 negative regulation of XA21-mediated resistance, we initiated in vitro biochemical studies. We first examined whether *Xb15* encodes a functional PP2C and if alterations in XA21-mediated resistance are caused by the putative PP2C activity of XB15. Full length XB15 (GST-XB15FL) tagged with an N-terminal tagged glutathione-S-transferase (GST) recombinant fusion protein and GST protein alone were expressed in *E*. *coli*. A smaller clone expressing the PP2C catalytic region alone, without the N-terminal extension (GST-XB15ΔN) was also expressed to check its possible regulatory function. We purified and assayed the recombinant proteins for phosphatase activity by measuring the release of phosphate from a phosphorylated synthetic peptide [57]. Figure 7A shows that in the presence of the substrate, both GST-XB15FL and GST-XB15ΔN, but not GST alone, catalyze the release of 240–350 pmoles of phosphate. This reaction was inhibited by the serine/threonine phosphatase inhibitor, sodium fluoride (NaF), but not by vanadate, a tyrosine phosphatase inhibitor. These results indicate that *Xb15* encodes a protein serine/threonine phosphatase not a tyrosine phosphatase, as predicted on the basis of a sequence analysis that places XB15 in the PP2C clade.

To experimentally test which class of serine/threonine phosphatases XB15 falls into, we incubated the recombinant GST-XB15FL, GST-XB15ΔN, and GST proteins with the substrate in various reaction buffers optimized for activity for different protein PP classes, PP2A, PP2B, and PP2C (Figure 7B) [57]. As predicted, XB15 enzyme activity was detected in PP2C buffer but not in PP2A and PP2B buffer. The presence of Mg^2+^ in the PP2C buffer was required for full activity. Significant inhibition was observed in PP2B buffer with saturating amounts of calmodulin and additional Ca^2+^. While GST-XB15FL showed a slightly lower PP activity than GST-XB15ΔN, unlike POL, inhibition of phosphatase activity by the N-terminal domain appears to be minimal (Figure 7A and 7B).

To test whether plant-expressed XB15 has PP2C activity, we used the NTAP-XB15 transgenic plants described above. The NTAP tag includes two IgG binding domains from Staphylococcus aurous protein A and a calmodulin binding peptide linked by a TEV protease cleavage site [51,52]. This tag has been successfully used for purification of native protein complexes from yeast, plants, and animals [52,58,59]. The NTAP-tagged XB15 (NTAP-XB15) and NTAP alone were purified with IgG-agarose and assayed for their phosphatase activity in PP2C buffer. Figure 7C shows that NTAP-XB15 purified from transgenic rice plants carries PP2C activity, but not purified NTAP alone or boiled NTAP-XB15. These experiments demonstrate that *Xb1*5 encodes a serine/threonine protein PP2C.

### XB15 Interacts with XA21 In Vitro and In Vivo

**Figure 8:**
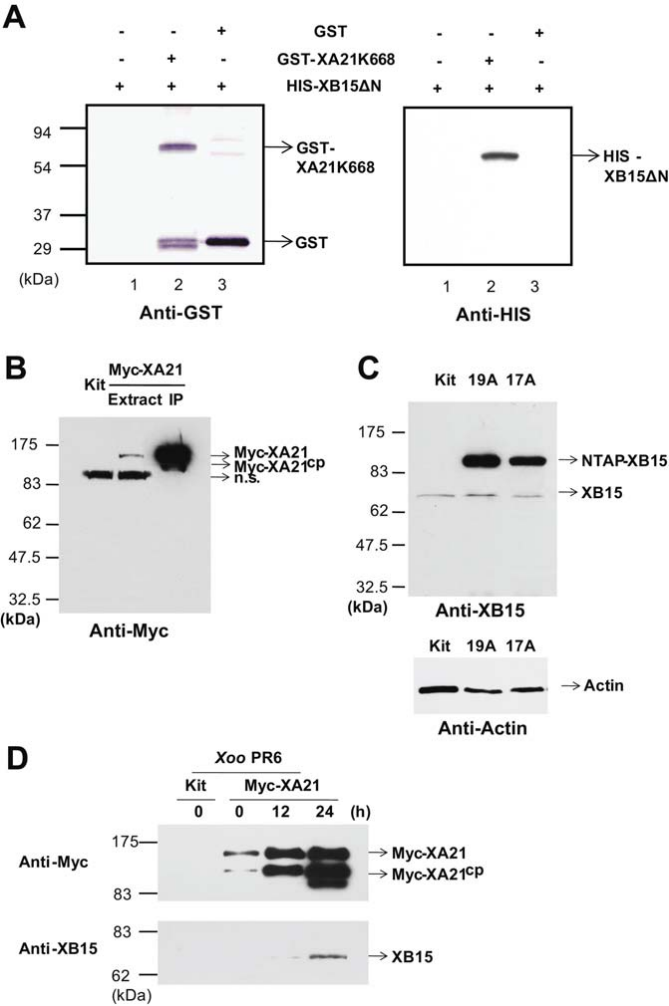
XB15 Interacts with XA21K668 In Vitro and In Vivo (A) HIS-XB15ΔN was incubated with either glutathione alone (lane 1), glutathione-bound GST-XA21K668 (lane 2), or GST (lane3) in buffer containing 2 mM MgCl_2_. After washing three times, proteins were eluted with 2× sample buffer, and analyzed by SDS-PAGE and immunoblotting with anti-GST or anti-HIS antibody. The experiment was repeated three times with similar results. (B) Immunodetection of Myc-XA21 protein in transgenic plants carrying Myc-Xa21 under control of its native promoter before (Extract) and after immunoprecipitation (IP). Myc-XA21 and Myc-XA21^cp^ give bands at approximately 140 and 100 kDa, respectively, corresponding to the predicted mass of each protein [42]. A nonspecific band (n.s.) of 95 kDa was detected. (C) Immunodetection of XB15 in Kitaake (Kit) and transgenic plants overexpressing NTAP-XB15 (17A and 19A). Anti-XB15 antibody detected at band of about 70 kDa of XB15 in Kit and of 95 kDa of in NTAP-XB15 in expressing plants (17A and 19A). Anti-actin antibody was used as a loading control. (D) Co-immunoprecipitation of XB15 and XA21 after *Xoo* PR6 inoculation. XA21 was detected after immunoprecipitation with agarose conjugated anti-Myc antibodies. Total protein extracts from 15 g of leaf tissue of Kitaake (Kit), mock-treated Myc-XA21, and *Xoo* PR6-inoculated Myc-XA21 at the indicated time points were incubated with anti-Myc antibody to immunoprecipitate XA21 complex. The precipitates were used for protein gel blot analysis using anti-Myc antibody (top) or anti-XB15 antibody (bottom). Myc-XA21 and Myc-XA21^cp^ give bands at about 140 and 100 kDa, respectively, and XB15 is detected as a band of 70 kDa.

XB15 was originally isolated as one of XA21 binding proteins using the yeast two-hybrid screen. To rule out the possibility of false positive caused by nonspecific binding to XA21, the specificity of the interaction between XA21 and XB15 was confirmed using a GST pull-down assay with immobilized GST-XA21K668. GST-XA21K668 and HIS-XB15ΔN were expressed in E. coli and purified using glutathione and Nichel nitrilotriacetic (Ni-NTA) agarose beads, respectively. The purified recombinant HIS-XB15ΔN protein was incubated with glutathione beads bound to either GST-XA21K668 or GST alone in a binding buffer containing 2 mM MgCl_2_. Figure 8A shows that the recombinant protein HIS-XB15ΔN specifically interacts with GST-XA21K668 (lane 2) but not GST (lane 3) or glutathione beads (lane 1).

To further investigate the association between XA21 and XB15 in vivo, we used the Myc-XA21 transgenic plants described above. Anti-Myc antibody detected a 140-kDa polypeptide in transgenic plants carrying *Myc-Xa21* but not in the control line Kitaake (Kit) (Figure 8B). When the Myc-XA21 protein is enriched after immunoprecipitation (IP) with agarose conjugated anti-Myc antibody, an additional 100-kDa product was detected. We have previously reported that the 140-kDa polypeptide is Myc-XA21 and the 100-kDa polypeptide is a proteolytic cleavage product of Myc-XA21 (Myc-XA21^cp^) [42]. We then developed a polyclonal antibody against a synthetic peptide of XB15 (anti-XB15) to detect the XB15 protein in planta and confirmed its specificity against XB15 from rice extracts. Anti-XB15 detected a major band of 70 kDa, which is close to the predicted size of XB15 (69.2 kDa) (Figure 8C). In the NTAP-XB15 overexpression line 17A and 19A, there was a significant increase in NTAP-XB15 (95 kDa) compared to endogenous XB15 (70 kDa).

When rice protein extracts were incubated with agarose conjugated anti-Myc antibody, an immune complex containing a 140-kDa polypeptide was precipitated and detected after western blot analysis with anti-Myc antibody in Myc-XA21 transgenic plants inoculated with *Xoo* PR6 but not in the Kitaake control (Figure 8D). Although the same amount of total protein extract was used for each immunoprecipitation, the amount of Myc-XA21 protein precipitated by anti-Myc-antibody accumulated to greater amounts at 12 and 24 h after *Xoo* PR6 inoculation as compared to the 0-h time point. Next, we examined whether XB15 was co-immunoprecipitated with XA21 in the immune complex. A 70-kDa polypeptide was detected with the anti-XB15 antibody in *Xoo* PR6-inoculated protein extracts. This band was not detected in Kitaake lacking *Myc-Xa21* and was barely detectable in mock-treated Myc-XA21 plants. The association between XB15 and XA21 was detectable by 12 h after inoculation with *Xoo* PR6 and had significantly increased after 24 h. As a control, similar experiments were performed with agarose-conjugated anti-Myc antibody presaturated with Myc peptide to check for a nonspecific interaction of XB15 with Myc peptide; we detected no interaction between XB15 and the Myc peptide (unpublished data). These results demonstrate a direct interaction between XB15 and XA21 in leaves, consistent with our in vitro observations.

### XA21 Is a Substrate of XB15

**Figure 9:**
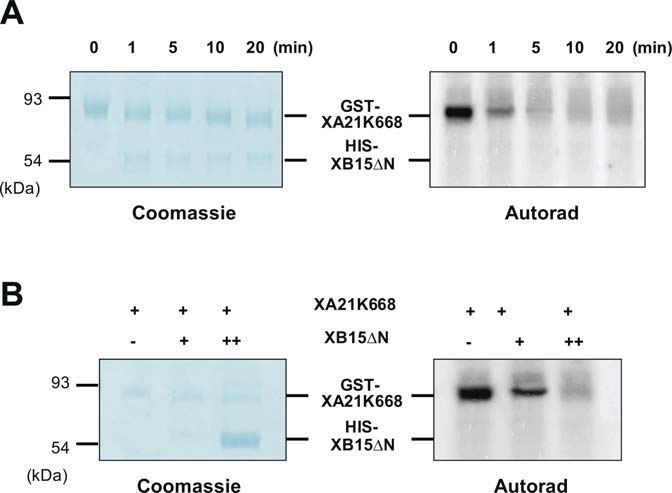
XB15 Dephosphorylates the XA21 Kinase Domain (A) Immobilized GST-XA21K668 was autophosphorylated with [γ-^32^P] ATP in kinase buffer. After washing, activated GST-XA21K668 was incubated with HIS-XB15ΔN (1 μg) at 37 °C for the indicated time (0, 1, 5, and 20 min). The samples were subsequently boiled in the presence of 2× sample buffer, separated by SDS-PAGE, stained with Coomassie brilliant blue R250 and destained (Coomassie). For the autoradiography, the dried gel was exposed to an image plate (Autorad). (B) Activated GST-XA21K668 was incubated with 1 μg (+) or 10 μg (++) of HIS-XB15ΔN at 37 °C for 5 min. The samples were subsequently boiled in the presence of 2× sample buffer, separated by SDS-PAGE, stained with Coomassie brilliant blue R250 and destained (Coomassie). For the autoradiography, the dried gel was exposed to an image plate (Autorad).

We have previously shown that XA21 encodes a protein kinase and that autophosphorylation of the XA21 kinase domain occurs via an intramolecular mechanism [43]. Because XB15 is associated with XA21 both in vitro and in vivo, because the overexpression of XB15 compromises the XA21-mediated resistance, and because XB15 possesses PP2C activity, we hypothesized that XB15 could directly dephosphorylate XA21. We tested this hypothesis by checking if XB15 could dephosphorylate the in vitro autophosphorylated XA21. After expression in E. coli and purification, GST-XA21K668 bound to glutathione beads was autophosphorylated with [γ-^32^P] in vitro. Autophosphorylation was monitored by radioactive incorporation of γ-^32^P-ATP into the bead-binding fraction (unpublished data) and by SDS-PAGE. The exposure to X-ray film shows that the fusion protein, GST-XA21K668 is capable of autophosphorylation (Figure 9A, 0 min).

The ^32^P-autophosphorylated GST-XA21K668 was incubated with or without 1 μg of purified recombinant HIS-XB15ΔN in the presence of PP2C buffer for the indicated time (Figure 9A). While XA21K668 remained labeled for over 60 min in the absence of XB15 (unpublished data), dephosphorylation of XA21K668 was detectable after 1 min of incubation with XB15. Additionally, dephosphorylation of XA21K668 by HIS-XB15ΔN was not detected in the presence of serine/threonine PP inhibitor, NaF (20 mM) or when HIS-XB15ΔN (1 μg) was boiled (unpublished data) suggesting that the dephosphorylation of XA21 is caused by the PP2C activity of XB15. In further support of this conclusion, increasing the amount of HIS-XB15ΔN (1–10 μg) resulted in a dosage-dependent dephosphorylation of GST-XA21K668 (Figure 9B). Although XA21-dependent phosphorylation of XB15 was examined with recombinant proteins purified from E. coli at various time points and different conditions, we did not detect transphosphorylation of XB15 by XA21 (unpublished data). From these results, we conclude that XA21 is a substrate of XB15 and that the XB15 phosphatase can effectively dephosphorylate XA21.

### An XB15-smGFP2 Fusion Protein Localizes to the Plasma Membrane

To further elucidate XB15 function, we assessed its subcellular localization. According to MultiLoc (http://www-bs.informatik.uni-tuebingen.de/Services/MultiLoc) [60] and PSORT (http://www.psort.org/) [61], XB15 has a putative nuclear-localization sequence (Figure 2) and is predicted to be a nuclear protein. To investigate the in vivo cellular distribution of XB15, a targeting experiment was performed using smGFP2, as a fluorescent marker [62]. We fused the entire *Xb15* coding region without the termination codon to the *smGFP2* gene (Figure S4A), and the resulting construct was introduced into rice Kitaake protoplast cells by PEG-mediated transformation [63,64]. Localization of the fusion protein was determined by visualization with an Olympus FV1000 confocal microscope. We introduced the *smGFP2* gene alone into protoplast cells as a control. As shown in Figure S4B, the fusion protein was mainly localized to the plasma membrane of the rice protoplast cell, whereas the control smGFP2 was uniformly distributed throughout the cell except the large central vacuole. As a positive control for plasma membrane targeting, we cotransfected protoplasts with vector expressing a H^+^-ATPase-red fluorescent protein (dsRed) fusion protein, which also localized to the plasma membrane [65]. A close overlap was observed between the green and red fluorescent signals of XB15-smGFP2 and H^+^-ATPase-dsRed, respectively. This experiment was repeated with protoplasts extracted from transgenic rice carrying *Xa21* gene and same localization of XB15 to the plasma membrane was observed (unpublished data).

We next tested if the large green fluorescent protein (GFP) tag would inhibit XB15 phosphatase activity. XB15-smGFP2 and smGFP2 alone were purified from transiently transformed protoplasts with agarose conjugated anti-GFP antibody and assayed for their phosphatase activity in PP2C buffer (Figure S5). We found that this purified XB15-smGFP2 carries PP2C activity similar to that observed for NTAP-XB15 (Figure 7C), but not purified smGFP2 alone or boiled XB15-smGFP2. Taken together, these results suggest that the tagged protein is functional and that, in vivo*,* XB15 is primarily targeted to the plasma membrane. Because its membrane targeting appears to be constitutive external signals are likely not required for its membrane translocation. This observation is also consistent with the predicted plasma membrane-localization of the intact XA21 protein [44].

## Discussion

Recognition of pathogen-associated molecules by PRRs is a critical component of innate immunity in animals and plants [66]. The rice XA21 protein is representative of a very large class of plant PRRs that carry the non-RD motif in the kinase domain [10]. The regulation of this important subclass of kinases has not yet been elucidated. Here, we have shown that rice employs a novel PP2C, XB15, to attenuate the XA21-mediated innate immune response.

### Functions and Subcellular Localization of XB15 Versus *Arabidopsis* POLs

XB15 possesses an active PP2C domain in its C-terminal region. On the basis of sequence comparison and phylogenetic analysis among PP2Cs from the rice and *Arabidopsis* databases, XB15 shows relatively high similarity with *Arabidopsis* POL, PLL4, and PLL5 (Figure 2B). All cluster into a small subgroup of the rice/*Arabidopsis* phylogenetic tree.

Abnormal leaf development was observed in *Arabidopsis* knockout mutants for *pll4* and *pll5* and transgenic plants overexpressing *Pll5* [48]. Despite the sequence similarity with POL and PLLs, we observed no disorders of leaf development in the *Xb15* knockout mutant and RNAi plants. Additionally, transgenic plants overexpressing *Xb15* displayed normal leaf development. These results indicate that XB15 has distinct functions to that of the *Arabidopsis* POL and PLLs.

The apparent differences in the function of XB15 and *Arabidopsis* POLs may be caused by their different localizations. *Arabidopsis* POL contains a putative nuclear-localization motif [47] and is predicted to be a nuclear protein according to the prediction methods for subcellular location, MultiLoc, and PSORT. In contrast, the fusion protein, XB15-smGFP2 is mainly localized to the plasma membrane of rice protoplast cells, suggesting that the in vivo function of XB15 is primarily related to the plasma membrane itself or to other membrane-localized proteins (Figure S4). Based on sequence analysis, XA21 is predicted to localize to the plasma membrane like other RKs, but its subcellular localization has not yet been demonstrated in vivo. Our in vivo data of XB15 combined with the putative localization of XA21 suggest that the interaction between XB15 and XA21 occurs at the plasma membrane, and may be similar to KAPP, which interacts with its target RKs at the plasma membrane to attenuate their signaling [34,36,67].

### Recruitment of XB15 to the JM Region of XA21

In animals, some receptor tyrosine kinases are controlled through autophosphorylation of their JM domains. For example, the mouse ephrin and KIT receptors, and human Fms (Feline McDonough Sarcoma)-like tyrosine kinase 3, interact with protein tyrosine phosphatases via phosphorylated tyrosine residues in their JM domains. These interactions negatively regulate receptor tyrosine kinase-mediated signaling [68–71]. In plants, only a few RKs have been shown to autophosphorylate residues in their JM regions. These include barley HvLysMR1, legume symbiosis RK*, Arabidopsis* BRI1, and rice XA21 [43,72–76]. In spite of the presumed importance of the JM domain in plant RK-mediated signaling, no downstream target proteins that bind the JM regions and regulate RK signaling in a phosphorylation-dependent manner have yet been identified.

XB15 failed to interact with the XA21K(TDG) mutant lacking the XA21 JM region and the catalytically inactive mutant XA21K668^K736E^ (Figure 1), suggesting that XA21 autophosphorylation in the JM region is critical for the XB15/XA21 interaction [43]. Although autophosphorylation of XA21 has not been demonstrated in yeast, there have been many reports showing that plant proteins maintain their function when they are expressed in yeast as heterologous proteins [77–79]. For example, an *Arabidopsis* kinase, GSK3/shaggy-like protein rescues the phenotype of its yeast homolog *mck1*, suggesting that plant kinases in yeast cells undergo the necessary post-translational modification for their in vivo functions [78]. Autophosphorylation of the JM residues Ser686, Thr688, and Ser689 was previously shown to be important stabilizers of XA21 protein levels [44]. XA21 proteins with mutations in these residues maintained interaction with Xb15, suggesting that these phosphorylation sites mainly function in the stability of XA21 protein but are not directly involved with mediating the downstream signal transduction cascade. In contrast, when Ser697 is mutated to Alanine, interaction with XB15 is abolished, indicating that Ser697 is essential for XA21 binding with XB15 in yeast. The Ser697 mutation does not affect kinase activity of XA21^S697A^, suggesting that Ser697 serves as a high affinity binding site rather than as a regulator of kinase activity (unpublished data, X. Chen, P.E. Canlas, M. Chern, D. Ruan, R. Bart, et al.). Taken together, these results suggest that XA21 requires its own kinase activity and residues in the JM region for interaction with XB15.

In support of this hypothesis, our preliminary results suggest that transgenic rice carrying the XA21 variant, XA21^S697A^, display enhanced resistance to *Xoo* PR6 at the 3-wk-old stage (unpublished data, X. Chen, P.E. Canlas, M. Chern, D. Ruan, R. Bart, et al.). These results indicate that Ser697 is essential for XA21 binding with XB15 and that in the absence of XB15 binding to XA21, negative regulation is compromised leading to enhanced resistance.

### XB15 Negatively Regulates Cell Death and the XA21-Mediated Innate Immune Response

In both animals and plants, PCD is a highly regulated process involved in immunity and other functions [66]. Many mutants exhibiting spontaneous PCD, initially isolated in maize [80], have now been identified in other plants, including *Arabidopsis*, barley, and rice [80–86]. Analysis of these mutants has led to the identification of genes that regulate cell death [87].

The *Xb15 Tos17* insertional mutant line, NF9014, and *Xb15* RNAi transgenic rice lines, RNAiXB15, display spontaneous cell death on the leaves during development under green house condition (26–28 °C) in the absence of obvious stress or disease. All of the tested defense-related *PR* genes commonly associated with the PCD and defense response [87–89] are constitutively expressed in the NF9014 mutant. In addition, progeny (F_4_) carrying both *Xa21* and the *Xb15 Tos17* mutation derived from the cross (NF9014–1/Myc-XA21) shows enhanced resistance to *Xoo* PR6, indicating that XA21-mediated resistance is enhanced in the absence of XB15 (Figure 6). In the absence of XA21, we do see enhanced resistance, although this difference is not statistically significant. We believe this unexpected result is due to the limitations in sensitivity of our assay.

The PCD-like phenotype observed in plants lacking *Xa21* suggests the presence of an additional signaling cascade that is negatively regulated by a functional XB15 (Figure 5). There are many examples of PP2Cs that function as negative regulators of multiple kinase cascades. In addition to *Arabidopsis* KAPP, which associates with at least five RKs [31–36], mouse PP2Cɛ dephosphorylates and negatively regulates MKKKs, apoptosis signal-regulating kinase 1 and transforming-growth-factor-β-activated kinase 1 (TAK1) [90]. In this report, we provide evidence that XB15 regulates at least one, but likely more, RK-mediated pathways, controlling PCD-like lesions.

### Model for XB15 Regulation of the XA21-Mediated Defense Response

**Figure 10:**
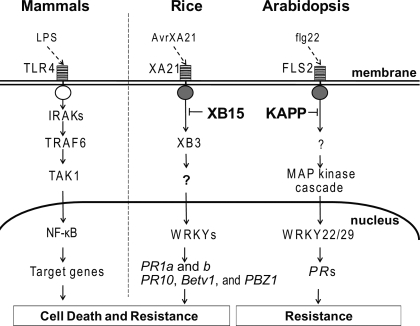
Comparison of Mammal, *Arabidopsis*, and Rice PRR-Mediated Signaling The LRR domain of TLR4 recognizes lipopolysaccharide signal (LPS) and transduces the signal across the cell membrane [91]. An adaptor molecule myeloid differentiation factor 88 (MyD88) associated with TLR4 recruits the non-RD serine/threonine kinase, IRAK1, which associates with tumor necrosis factor receptor associated factor 6 (TRAF6) in cytoplasm [102]. TRAF6 forms a complex with TAK1 [93] in which the kinase activity of, TAK1 mediates downstream events, such as inhibitor of κ kinase complex and NF-κB [5,95]. In *Arabidopsis*, flg22 binds to FLS2, which transduces the pathogen signal through its cytoplasmic non-RD kinase domain, and activates MAPK cascade [15]. Several WRKY transcription factors acting downstream of MAPK cascade to induce defense-related genes including PR genes [97]. KAPP, a PP2C, blocks the activated FLS signaling and attenuates the downstream innate immune response [33]. In rice, AvrXA21 binding to XA21 activates its cytoplasmic non-RD kinase [18,20]. XA21 transphosphorylates proteins such as XB3, which are required for the defense response [42]. In the nucleus, WRKY transcription factors activate defense-related genes such as *PR1* and *PR10* of XA21 signaling pathway. Recruitment of XB15 to Ser697 in the XA21 JM domain and subsequent dephosphorylation of phosphorylated residue(s) attenuates XA21 signaling.

Figure 10 compares our working model for XA21-mediated innate immunity with the human TLR4 and *Arabidopsis* FLS2 signaling cascades. TLR4 contains an extracellular LRR that is critical for transmitting the lipopolysaccharide signal (LPS) across the cell membrane [91]. An adaptor molecule, myeloid differentiation factor 88 (MyD88), associated with the Toll/interleukin-1 receptor (TIR) intracellular domain of TLR4, recruits IRAK4 [5]. The activated IRAK4 rapidly phosphorylates the non-RD kinase IRAK1, and leaves the receptor complex to interact with the tumor necrosis factor receptor associated factor 6 (TRAF6) [92]. TRAF6 is autoubiquitinated and forms a complex with TAK1, which functions as a MAPK kinase kinase [93,94]. The activated kinase TAK1 mediates downstream events, such as the activation of inhibitor of κ kinase, p38, and Jun-N-terminal kinases, which lead to the activation of transcription factors including NF-κB and activating protein-1 [5,95].

Similar to TLR4, *Arabidopsis* FLS2 also carries an extracellular LRR domain that recognizes a pathogen-associated molecule; in this case a small peptide called flg22 [15]. FLS2 contains an intracellular non-RD kinase, which, when activated, transduces the signal to a MAPK cascades [96–98]. WRKY transcription factors in the nucleus are activated by the MAPK cascades and turn on downstream target genes [97]. KAPP has been shown to negatively regulate FLS2-mediated signaling through overexpression studies, although the mechanism has not yet been elucidated [33].

In rice, the XA21 LRR domain is responsible for race-specific recognition of *Xoo* strains carrying AvrXA21 [20,99]. XA21/AvrXA21 binding is hypothesized to activate the non-RD kinase domain leading to XA21 autophosphorylation and/or transphosphorylation of downstream target proteins [20,100]. In support of this hypothesis we have observed a strong interaction between XB15 and XA21 in *Xoo* PR6-inoculated rice plants but not in untreated rice (Figure 8D), suggesting that *Xoo* inoculation induces XA21/XB15 complex formation. Our data indicate that Ser697 in the XA21 JM region is required for XB15 binding.

XA21 transphosphorylates the RING finger ubiquitin ligase XB3, which is required for effective XA21-mediated resistance [41,92]. We have also shown a direct, regulatory role of XB10 (OsWRKY62) in the XA21-mediated response [41], suggesting another layer of conservation between the *Arabidopsis* FLS2 and rice XA21 signaling pathways. In the nucleus, XB10 and other WRKY transcription factors (unpublished data, Y. Peng, L.E. Bartley, and P.C. Ronald) [41] either activate or repress defense-related genes such as *PR1* and *PR10*.

The XA21-mediated defense response is also regulated by rice negative regulator of resistance (NRR) that interacts with NH1 [53,54], the rice ortholog of NPR1, a key regulator of the SA-mediated defense pathway (unpublished data). This result demonstrates cross-talk between the XA21- and NH1-mediated pathways.

In contrast to animal PPs that inactivate phosphorylated MAPKs to negatively regulate the innate immune response [24], XB15 directly interact with the non-RD kinase domain of XA21. This study suggests that XA21 is a substrate of XB15 and that the phosphatase activity of XB15 attenuates the XA21-mediated innate immune response. Future studies will be directed at identifying the XA21 phosphorylated residue(s) targeted by XB15 for dephosphorylation.

## Materials and Methods

### Plant material and growth conditions.

Rice (Oryza sativa L.) plants were maintained in the green house. The growth chamber was set on a 16-h light and 8-h dark photoperiod, a 28/26 °C temperature cycle, and 90% humidity. Healthy and well-expanded leaves from 6-wk-old rice plants were used for *Xoo* PR6 inoculation and nucleic acid or protein extraction. XA21K668 and XA21K(TDG) were amplified using the primer pairs 5′-CACCATGTC ATCACTCTACTTGCTTA-3′/5′-TCAGAATTCAAGGCTCCCA-3′ and 5′-CACCATGACAGATGGTTTCGCGCCGACC-3′/-TCAGAATTCAAGGCTCCCA-3′, respectively, and cloned into pENTR/D-TOPO/D vector (Invitrogen). XA21K668^K736E^, XA21K668^S686A^, XA21K668^T688A^, XA21K668^S689A^, XA21K668^S686A,T688A,S689A^, and XA21K668^S697A^ were constructed by site-directed mutagenesis using the Quick Change kit (Stratagene), according to the manufacturer's protocols. The specific primers for the mutagenesis were 5′- GTTGCAGTGGAAGTACTAAAGCTTGAAAATCC-3′/5′- GGATTTTCAAGCTTTAGTACTTCCACTGCAAC-3′ (for XA21K668^K736E^), 5′- AAGGGAGCCCCTGCAAGAACTTCCATG-3′/5′- CATGGAAGTTCTTGCAGGGGCTCCCTT-3′ (for XA21K668^S686A^), 5′- GCCCCTTCAAGAGCTTCCATGAAAGGC-3′/5′- GCCTTTCATGGAAGCTCTTGAAGGGGC-3′ (for XA21K668^T688A^), 5′- CCTTCAAGAACTGCCATGAAAGGCCAC-3′/5′- GTGGCCTTTCATGGCAGTTCTTGAAGG-3′ (for XA21K668^S689A^), 5′- AAGGGAGCCCCTGCAAGAGCTGCCATGAAAGGCCAC-3′/5′- GTGGCCTTTCATGGCAGCTCTTGCAGGGGCTCCCTT-3′ (for XA21K668^S686A,T688A,S689A^), and 5′- CACCCATTGGTCGCTTATTCGCAGTTG-3′/5′-CAACTGCGAATAAGCGACCAATGGGTG-3′ (for XA21K668^S697A^). The positive clones were verified by DNA sequencing and then using Gateway LR Clonase (Invitrogen), moved into the yeast two-hybrid vector pNlexA carrying the BD domain (Clontech). Positive clones were confirmed again by DNA sequencing. AD-Xb15 was from the clone pAD-GAL4–2.1 identified from a cDNA library. The purified plasmid DNAs from AD vectors and BD vectors were cotransformed into the yeast cell pEGY48/p8op-LacZ (Clontech) using the Yeast transformation kit, Frozen-EZ yeast transformation II (Zymo Research). We followed the detailed procedure from the manual of Matchmaker LexA Two-Hybrid System (Clontech).

### Production of recombinant protein.

Full-length cDNA corresponding to *Xb15* was amplified by PCR with primers 5′-CACCATGGGCAACTCCCTCGCCTG-3′/5′-TTACACGCAGGATCTCCAAATC-3′. PCR fragments were purified and subcloned into the pDEST15 or 17 (Invitrogen), which expresses the recombinant protein with an N-terminal GST or HIS tag, respectively. The resulting expression vectors were transformed into the bacterial host strain BL21 (DE3) pLysS (Invitrogen), and expression of protein was induced at midlog phase (1 mM isopropyl β-D-thiogalactosiadse, 3 h, 28 °C). Recombinant proteins were purified by affinity chromatography using Glutathione Sepharose 4B (Amersham) or Ni-NTA bead (Qiagen).

For the purification of the recombinant protein from rice, leaves of 5- to 6-wk-old NTAP-XB15 transgenic plants were harvested essentially as previously described [51,52]. Five grams fresh weight of rice shoots were ground to a fine powder in liquid nitrogen. Crude protein extracts were prepared in four volumes of Extraction Buffer I (20 mM Tris-HCl, [pH 8.0], 150 mM ethylene-diamine-tetra-acetic acid (EDTA), 2 mM benzamidine, 0.1% IGEPAL, 10 mM β-mercaptoethanol, 20 mM NaF, 1 mM phenylmethanesulfonylfluoride [PMSF], 1% Protease cocktail (Sigma), 2 μg/ml leupeptin, 2 μg/ml antipain, and 2 μg/ml aprotinin). The extract was passed through a fine sieve, filtered through two layers of miracloth, and centrifuged twice at 13,000 g for 30 min at 4 °C. The cleared supernatant was mixed with 50 μl of IgG Sepharose beads (Amersham) and incubated at 4 °C for 2 h on Labquake Rotisserie (Barnstead Thernolyne). After centrifugation at 3,000 g for 30 sec, IgG supernatant was discarded and the collected IgG beads were washed four times in 5 ml Extraction Buffer I lacking protease inhibitors and twice in 1 ml of 5 mM ammonium acetate (pH 5.0). The protein was eluted with 1 ml of 0.5 M acetic acid (HOAc) (pH 3.4), neutralized with 100 μl of 1 M Tris-HCl (pH 8.0), and concentrated by acetone precipitation. The purified NTAP-XB15 protein was used for PP activity assay and protein gel blot analysis.

### In vitro GST pull-down assays.

Purified recombinant proteins, GST-XA21K668, HIS-XB15, and GST were used for pull-down assays as described [101], except that buffers were supplemented with 2 mM MgCl_2_. Bound proteins were eluted by boiling in SDS sample buffer, separated by SDS-PAGE, and detected by immunoblotting with a anti-histidine antibody (Quiagen). Recombinant GST or GST-XA21K668 were incubated with glutathione-sepharose beads (Amersham) overnight at 4 °C and washed three times for 10 min at 4 °C with phosphate buffer saline (PBS). The beads were equilibrated with Binding Buffer (20 mM Tris [pH 7.5], 150 mM NaCl, 2 mM MgCl_2_, 0.5 mM dithiothreitol) to which a protease inhibitor mixture (Roche) was added. Recombinant HIS-XB15 was incubated with GST or GST-XA21K668 (20 μg) bound to glutathione beads. After 2 h of incubation at 4 °C, the beads were washed four times for 20 min in binding buffer. Proteins bound to the bead were eluted with SDS sample buffer, separated by SDS-PAGE, and processed for immuno-blotting.

### In vivo co-immunoprecipitation.

To co-immunoprecipitate Myc-XA21 and XB15, total proteins were extracted from 5 g of leaf tissue in 25 ml of ice-cold Extraction Buffer II (0.15 M NaCl, 0.01 M Na-phosphate [pH 7.2], 2 mM EDTA, 1% Triton X-100, 10 mM β-mercaptoethanol, 20 mM NaF, 1 mM PMSF, 1% Protease cocktail [Sigma], 2 μg/ml leupeptin, 2 μg/ml antipain, and 2 μg/ml aprotinin). After filtering through Miracloth (Calbiochem) followed by centrifugation twice at 13,000 g for 20 min at 4 °C, the supernatant was mixed with 50 μl of agarose conjugated anti-Myc antibody (Santa Cruz) and incubated at 4 °C for 2 h. The beads were then washed four times in 1 ml of Extraction Buffer II without proteinase inhibitors. The proteins were eluted with 4× Laemmli loading buffer. Protein blot analyses were performed. To co-immunoprecipitate XB15-smGFP2, total proteins were extracted from protoplasts transformed with *Xb15-smGFP2* in 5 ml of ice-cold Extraction Buffer II and 20 μl of agarose conjugated anti-GFP antibody (Santa Cruz). For the anti-XB15 antibody, synthetic peptides and monospecific antibodies were made as a service by Pacific Immunology. Detailed information about their methods can be obtained at Pacific Immunology (http://www.pacificimmunology.com/). Epitope selection was directed to hydrophobic, flexible regions of the proteins. The epitope, TRALLARTEKFQDSADL was used. Peptides were synthesized and conjugated to keyhole limpet hemocyanin. Rabbits were immunized with peptide in complete Freund's adjuvant, followed by three boosts in incomplete Freund's adjuvant. Monospecific antibodies were affinity purified to the synthetic peptides bound to C3-SEP-PAK cartridges. Antibodies were conjugated to horseradish peroxidase for use in Western blots.

### *Xoo* inoculation and determination of bacterial populations.

For *Xoo* inoculation, rice plants were grown in the greenhouse normally until they were 6 wk old, unless stated otherwise, and transferred to the growth chamber. The *Xoo* strain Philippine race 6 (PR6) was used to inoculate rice by the scissors-dip method [18,53]. Only the top two to three expanded leaves of each tiller were inoculated. For *Xoo* colony counts from inoculated leaves, 20 cm of leaf tissue from the top, including lesions and tissue showing no lesions, was ground up and resuspended in 10 ml water to harvest bacteria. The extract was diluted accordingly and plated out on peptone sucrose agar (PSA) plates containing 15 mg/l cephalexin.

### Crossing with NTAP-XB15 and Myc-XA21.

The transgenic lines Myc-XA21 T330-16-1 was used as the pollen recipient in a cross with pollen donor NTAP-XB15 17A and 19A. Over 50 seeds were recovered from the each T330-16-1/17A and T330-16-1/19A cross. The nature of the F_1_ hybrid was confirmed by PCR amplification of 820-bp fragment spanning part of the *Myc* tag and *Xa21* in the *Myc-Xa21* construction using primers 5′-GAGCAAAAGCTGATTTCTGAGGAGGAT-3′/5′-ACCACCTAGCTTGTTTTCTCTGAC-3′ and 650-bp fragment spanning part of the *NTAP* tag and *Xa21* in the *Ntap-Xb15* construction using primers 5′-ATGCCCAAGCCCCAAAGGACTACG-3′/5′-GAAGCTTGGACGGCGCCACCCATACGAC-3′.

### Rice transformation.

Rice transformation was constructed as described previously [53]. Agrobacterium EHA105 was used to infect rice callus for transformation. Transformants of rice cultivars Kitaake carrying *Ntap-Xb15* or *Myc-Xa21* were selected using hygromycin.

### Immunodetection.

For NTAP-XB15 detection, rabbit PAP soluble complex (Sigma) was used at a final dilution of 1:5,000 for 2 h. Bands were visualized using the SuperSignal West Pico Chemiluminescent Substrate (Pierce) according to standard protocol.

### XB15-smGFP2 protein construction and transient expression.

PCR was performed using the gene-specific oligonucleotide primers, 5′-GGGATCCAATGGGCAACTCCCTCGCCTG-3′/5′-GGGATCCACGCAGGATCTCCAAATC-3′. Subsequently, the termination codon of the *Xb15* cDNA was removed. The PCR-amplified product was fused in-frame to the coding region of soluble-modified green fluorescent protein (smGFP2) (kindly provided by K.H. Paek) [63,64]. Transient expression of green fluorescent protein (GFP) fusion constructs and H^+^-ATPase-dsRed (kindly provided by I. Hwang) were performed by introducing the plasmid into the rice protoplasts using the PEG-mediated transformation method [63,64].

Images were collected with an Olympus FV1000 confocal microscope. GFP was imaged under the following conditions: excitation: 488 nm; DM 405/488/543; emission: 500–530 nm. DsRed was imaged under the following conditions: excitation: 543 nm; DM 405/488/543; emission: 560–620 nm. Images were collected through a 40× (NA: 1.00) oil immersion lens. All images are the result of 2 kalman line averages, and, where appropriate, sequential scans were used to prevent cross talk. Images were analyzed using the Olympus Fluorview software (Ver 1.4a) and coded green (for GFP) or red (for dsRed).

### RT-PCR.

For RT-PCR analysis, total RNAs were extracted from leaves after each treatment and then the RT reaction was performed following the manual for QuantumRNA 18S Internal Standards (Ambion). PCR analyses were performed with primers pairs, 5′-TTATCCTGCTGCTTGCTGGT-3′/5′-GGTCGTACCACTGCTTCTCC-3′ (for *PR1a*), 5′-AGGTATCCAAGCTGGCCATT-3′/5′-GGCGTAGTCGTAGTCGCTCT-3′ (for *PR1b*), 5′-CGCAGCTCACATTATCAAGTCAGA-3′/5′-GAAGCAGCAATACGGAGATGGATG-3′ (for *PR10*), 5′-GCAGGGAGCGTATACAAGACCAA-3′/5′-CACGCCACAGTAACATGACCACAA-3′ (for Betv1), 5′-CAACAGTCGAAGGGCAATAATAAGTC-3′/5′-ACTGCCACACCTCCCACATTG-3′, 5′-ATGGCTCCGGCCTGCGTCTCCGA-3′/5′-GGCATATTCGGCAGGGTGAGCGA-3′ (for *PBZ1*), 5′-TCATTCGATGGATCAGTCGGG-3′/5′-ATGCTCTGGTCACCTTCAGCG-3′ (for *Xb15*), and 5′-CAACAGTCGAAGGGCAATAATAAGTC-3′/5′-ACTGCCACACCTCCCACATTG-3′ (for *EF1α*). The amplified products were then resolved by gel electrophoresis.

### PP2C activity assay.

Phosphatase activity was measured according to the instructions provided by the manufacturer by using a nonradioactive serine/threonine phosphatase assay system (Promega). The color was allowed to be developed for 15 min, and the absorbance was measured at 600 nm with plate reader (Bio-Rad). The composition of the buffers used in the assay was: PP2A 5× buffer (250 mM imidazole [pH 7.2], 1 mM EGTA, 0.1% 2-mercaptoethanol, 0.5 mg/ml BSA), PP2B 5× buffer (250 mM imidazole [pH 7.2], 1 mM EGTA, e50 mM MgCl_2_, 2 mM CaCl_2_, 250 μg/ml calmodulin, 0.1% 2-mercaptoethanol), PP2C 5× buffer (250 mM imidazole [pH 7.2], 1 mM EGTA, 25 mM MgCl_2_, 0.1% 2-mercaptoethanol, 0.5 mg/ml BSA).

### In vitro phosphatase assays.

Purified agarose-bound fusion proteins, GST-XA21K668, were washed with kinase buffer (50 mM HEPES [pH 7.4], 10 mM MgCl_2_, 10 mM MnCl_2_, 1 mM dithiothreitol). Autophosphorylation experiments were carried out in 30-μl volumes containing 20 μl of agarose-bound protein (5 μg) and 20 μCi of [γ-^32^P]ATP (6,000 Ci/mmol) (PerkinElmer Life Science). The reaction was stopped after 30 min by adding 10 μl of Laemmli loading buffer and boiling for 5 min. The proteins were separated by SDS-PAGE (7.5% or 10%). After staining with Coomassie Brilliant Blue G-250, the gel was dried and exposed to x-ray film.

To dephosphorylate the phosphorylated fusion proteins by HIS-XB15ΔN, the ^32^P-labeled XA21K668 proteins were washed with PP2C buffer and incubated with HIS-XB15ΔN. The resulting proteins were resolved by SDS-PAGE as described above.

### Plasmid construction for *Xb15* overexpression.

A 1,920-nt cDNA fragment encoding full-length XB15 protein was amplified from a rice cDNA using primers, 5′-CACCATGGGCAACTCCCTCGCCTG-3′/5′-TTACACGCAGGATCTCCAAATC-3′. The PCR product was cloned into pENTR/D-TOPO (Invitrogen) according to the instructions provided by the manufacturer and the insert confirmed by sequencing. For Overexpression in rice, the *Xb15* cDNA in pENTR/D-TOPO was recombined into the final Ubi-NTAP-1300 vector using Gateway LR Clonase (Invitrogen). Ubi-NTAP-1300 is a pCAMBIA-1300 (AF234296) derivative with an additional expression cassette containing the maize ubiquitin promoter and a nopaline synthase 3′-polysdenylation region, to which the NTAP/Gateway cassette was added [52].

## Supporting Information

Figure S1 Immunodetection of Myc-XA21 in Homozygous and Heterozygous Myc-XA21 Lines Equal amounts (100 μg) of total protein form Kitaake wild type (Kitaake) and homozygous Myc-XA21 (F_3_, 19A-72-5-1 and 17A-18-1-1) and heterozygous Myc-XA21 (F_1_, 17A-24 and 19A-74) extracted and analyzed by SDS-PAGE, and immunoblotted with anti-Myc. Equal loading of total proteins was confirmed by immunodetection of actin protein with anti-actin antibody. Myc-XA21 gives bands at approximately 140 and a nonspecific band (n.s.) of 95 kDa was detected. Homo, homozygous for *Myc-Xa21*; Hetero, heterozygous for *Myc-Xa21*.
(3.18 MB TIF)

Figure S2 Homozygous and Heterozygous Progeny (M_5_) of *Xb15 Tos17* Insertion Mutant Lines (NF9014-6-16–30) Display Moderate to Severe Necrotic Lesions The number below the bar represents individual segregating progeny of NF9014-6-16–20. Level 1 indicates no lesion formation. Level 2 indicates a moderate number of lesions (approximately 20–40 lesions per leaf), and level 3 indicates the presence of many lesions (approximately 40–100 lesions per leaf, lesions open spread along the veins without separation, merging with nascent lesions). Six of the heterozygotes were used for expression analysis before observation of cell death lesions and are therefore not included in the figure.
(1.10 MB TIF)

Figure S3 An *RNAiXb15* Mutant Line Exhibits Cell Death (A) Phenotype of the *Xb15* RNAi line (RNAiXB15 4B-12) and the Kitaake control. Photograph was taken 6 wk after germination.
(B) Immunodetection of XB15 in *Xb15* RNAi line (RNAiXB15 4B) and Kitaake (Kit). Anti-XB15 antibody detected a band of about 70 kDa corresponding to XB15. Anti-actin antibody was used to detect equal loading of proteins in the two lanes.
(2.67 MB TIF)

Figure S4 XB15-smGFP2 Fusion Protein Is Localized to the Plasma Membrane (A) Construction of *Xb15-smGFP2* fusion gene. The PCR-amplified product without termination codon was fused in-frame to the coding region of smGFP2 [63,64]. Ubi pro, ubiquitin promoter; NOS ter, nopaline synthase terminator.
(B) The *Xb15-smGFP2* fusion, *H^+^-ATPase-dsRed* fusion, and control *smGFP2* constructs were introduced into rice protoplast cells by PEG-mediated transformation [63]. H^+^-ATPase-dsRed was used as a marker for plasma membrane protein. Expressions of the introduced genes were observed 16 h after transformation. Images were collected with an Olympus FV1000 confocal microscope. The images were coded green (for smGFP2) or red (for dsRed). Scale bar, 10 μm.
(4.98 MB TIF)

Figure S5 XB15-smGFP2 Is Functional Protein Possessing PP2C Catalytic Activity XB15-smGFP2 fusion protein and smGFP2 alone were purified from protoplasts transformed with each construct. Approximately 1 μg of each protein was incubated with 200 μM substrate in PP2C buffer for 60 min. For the boiled XB15-smGFP2, one μg of XB15-smGFP2 was boiled in water for 20 min. The data are average value of three experiments. Error bars represent standard deviations.
(410 KB TIF)

### Accession Numbers

The rice cDNA locus identification numbers (TIGR; http://rice.tigr.org/) are as follows: *Xb15*, Os03g60650; *PR1a*, Os07g03710; *PR1b*, Os01g28450; *PR10*, Os12g36830; *Betv1*, Os12g36850; *PBZ1*, Os12g36880; *EF1s*, Os03g08010. The GenBank (http://www.ncbi.nlm.nih.gov/Genbank) accession number for *Xb15* cDNA sequence is NP_001051726.

## Abbreviations

18S rRNA: 18S ribosomal RNA
Avr: avirulence
*Betv1*: *Betula verrucosa 1*
cfu/leaf: colony-forming units per leaf
DAI: days after inoculation
GFP: green fluorescent protein
IRAK: interleukin-1 receptor-associated kinase
KAPP: kinase-associated protein phosphatase
JM: juxtamembrane domain
LRR: leucine rich repeats
MAPK: mitogen-associated protein kinase
NTAP: N-terminal tandem affinity purification
PAP: peroxidase-anti-peroxidase
*PBZ1*: probenazole-inducible protein 1
PCD: programmed cell death
PLL: POL-like protein
PP: protein phosphatase
PP2C: protein phosphatase 2C
PRR: pathogen recognition receptor
RK: receptor kinase
RNAi: RNA interference
RT-PCR: reverse transcription-PCR
TAK1: transforming-growth-factor-β-activated kinase 1
TLR: Toll-like receptor
XB15: XA21 binding protein 15
*Xoo* PR6: *Xanthomonas oryzae* pv. *oryzae* Philippine race 6
