# Correction: Rice XB15, a Protein Phosphatase 2C, Negatively Regulates Cell Death and XA21-Mediated Innate Immunity

**DOI:** 10.1371/journal.pbio.0060282

**Published:** 2008-11-25

**Authors:** Chang-Jin Park, Ying Peng, Xuewei Chen, Christopher Dardick, DeLing Ruan, Rebecca Bart, Patrick E Canlas, Pamela C Ronald

Correction for:

Park CJ, Peng Y, Chen X, Dardick C, Ruan D, et al. (2008) Rice XB15, a protein phosphatase 2C, negatively regulates cell death and XA21-mediated innate immunity. PLoS Biol 6(9): e231 doi:10.1371/journal.pbio.0060231

The fourth author, Christopher Dardick's, affiliation is incorrectly listed as “Agricultural Research Service, Appalachian Fruit Research Station, United States Department of Agriculture, Kearneysville, West Virginia, United States of America.” It should be listed as “Department of Plant Pathology, University of California Davis, Davis, California, United States of America.” His current address is, “Agricultural Research Service, Appalachian Fruit Research Station, United States Department of Agriculture, Kearneysville, West Virginia, United States of America.”

Additionally, the licence used for this manuscript is incorrect. The correct licence statement for this article is:

**Copyright:** © 2008 Park et al. This is an open-access article distributed under the terms of the Creative Commons Attribution License, which permits unrestricted use, distribution, and reproduction in any medium, provided the original author and source are credited.

