# Supplementary figures and images for: Rice XB15, a Protein Phosphatase 2C, Negatively Regulates Cell Death and XA21-Mediated Innate Immunity
Source: PLoS Biol. 2008 Sep 23;6(9):e231. doi: 10.1371/journal.pbio.0060231 (PMC2553837; doi:10.1371/journal.pbio.0060231)

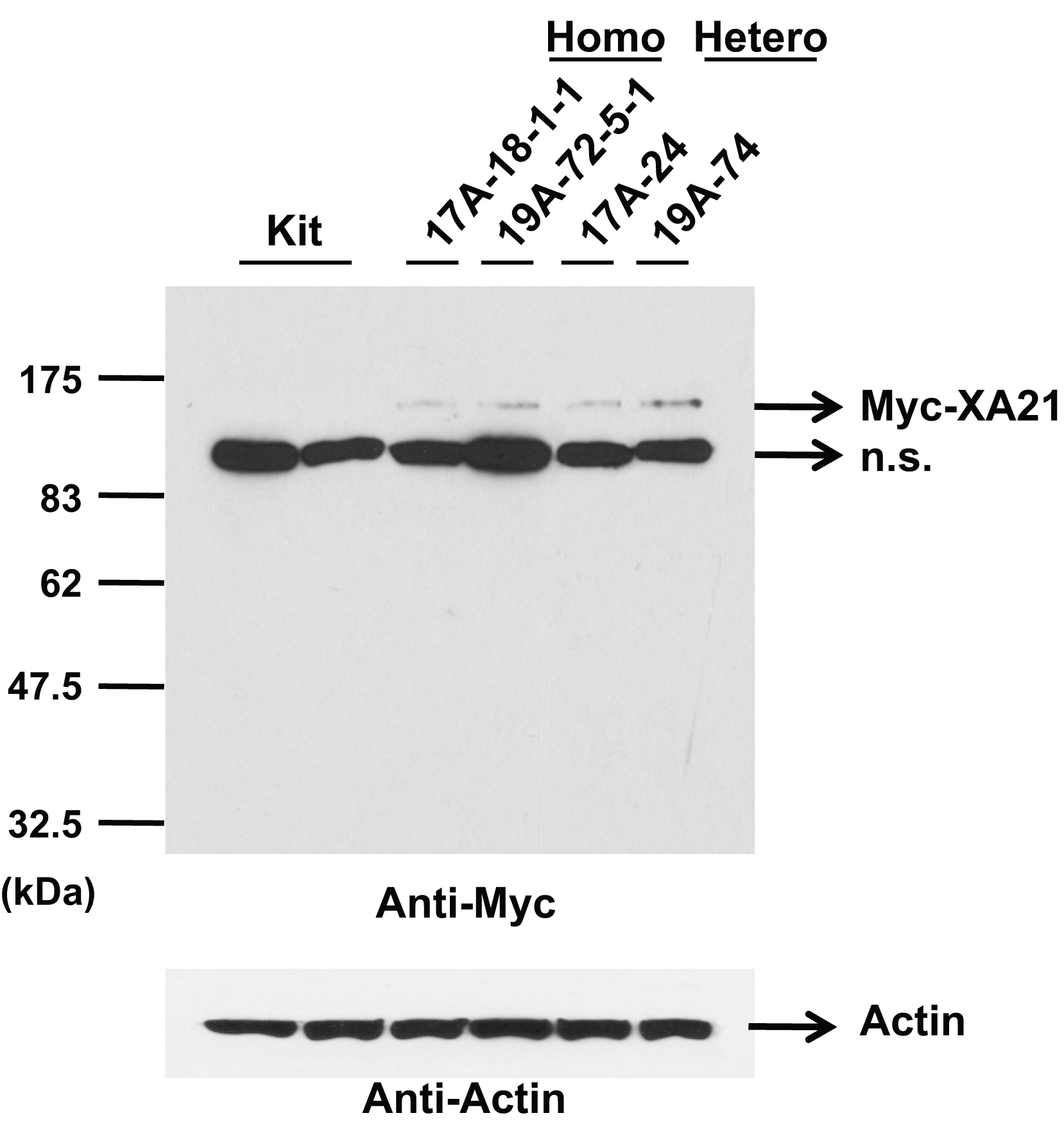

Supplement: Figure S1 — Equal amounts (100 μg) of total protein form Kitaake wild type (Kitaake) and homozygous Myc-XA21 (F3, 19A-72-5-1 and 17A-18-1-1) and heterozygous Myc-XA21 (F1, 17A-24 and 19A-74) extracted and analyzed by SDS-PAGE, and immunoblotted with anti-Myc. Equal loading of total proteins was confirmed by immunodetection of actin protein with anti-actin antibody. Myc-XA21 gives bands at approximately 140 and a nonspecific band (n.s.) of 95 kDa was detected. Homo, homozygous for Myc-Xa21; Hetero, heterozygous for Myc-Xa21. (3.18 MB TIF) [file pbio.0060231.sg001.tif]

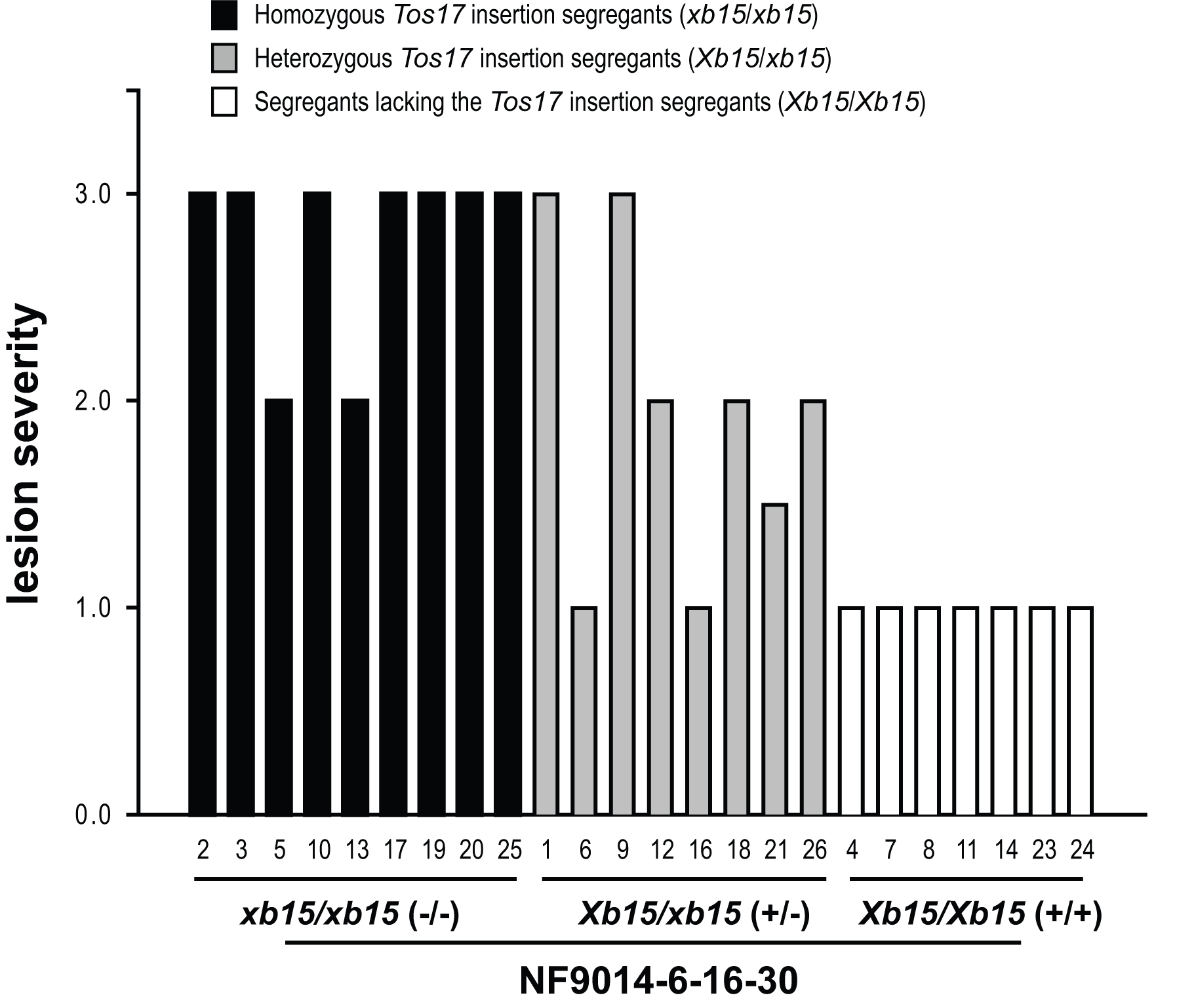

Supplement: Figure S2 — The number below the bar represents individual segregating progeny of NF9014-6-16–20. Level 1 indicates no lesion formation. Level 2 indicates a moderate number of lesions (approximately 20–40 lesions per leaf), and level 3 indicates the presence of many lesions (approximately 40–100 lesions per leaf, lesions open spread along the veins without separation, merging with nascent lesions). Six of the heterozygotes were used for expression analysis before observation of cell death lesions and are therefore not included in the figure. (1.10 MB TIF) [file pbio.0060231.sg002.tif]

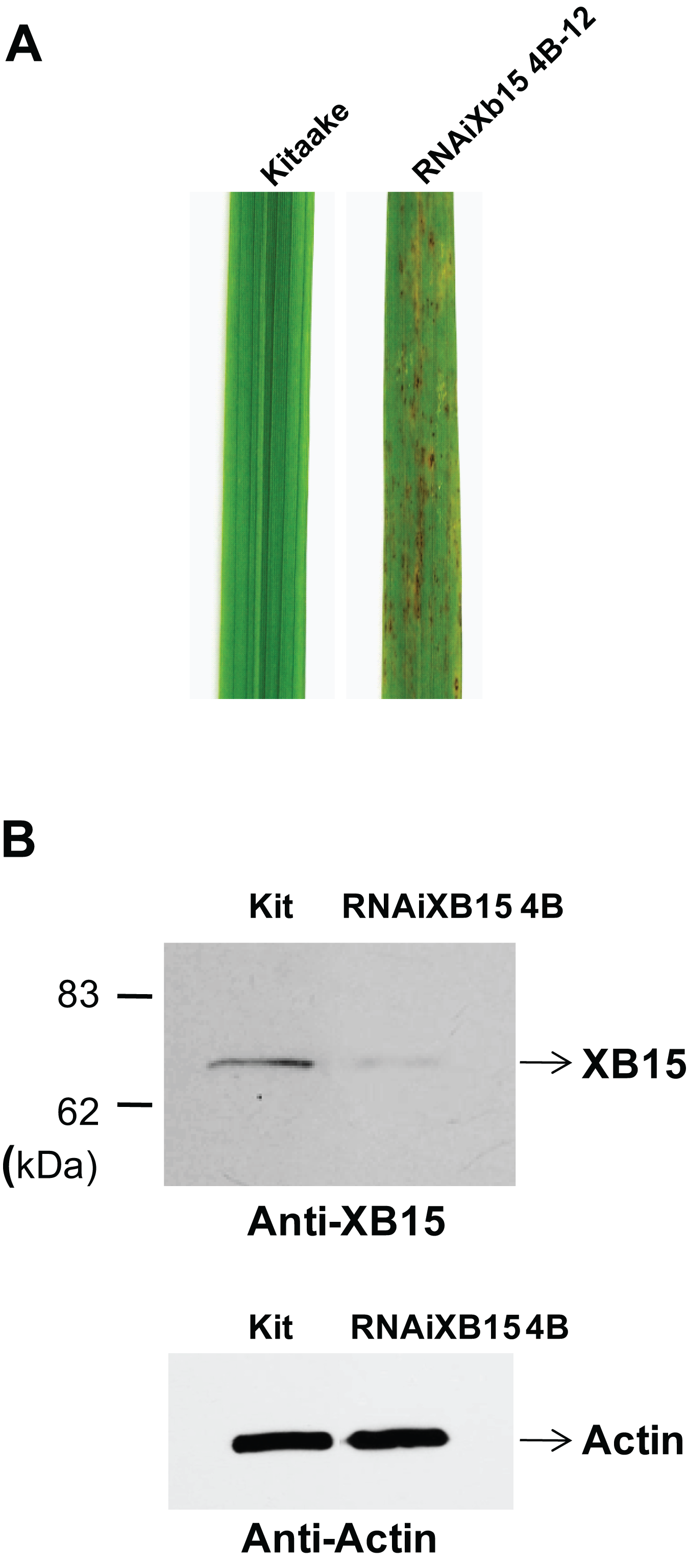

Supplement: Figure S3 — (A) Phenotype of the Xb15 RNAi line (RNAiXB15 4B-12) and the Kitaake control. Photograph was taken 6 wk after germination. (B) Immunodetection of XB15 in Xb15 RNAi line (RNAiXB15 4B) and Kitaake (Kit). Anti-XB15 antibody detected a band of about 70 kDa corresponding to XB15. Anti-actin antibody was used to detect equal loading of proteins in the two lanes. (2.67 MB TIF) [file pbio.0060231.sg003.tif]

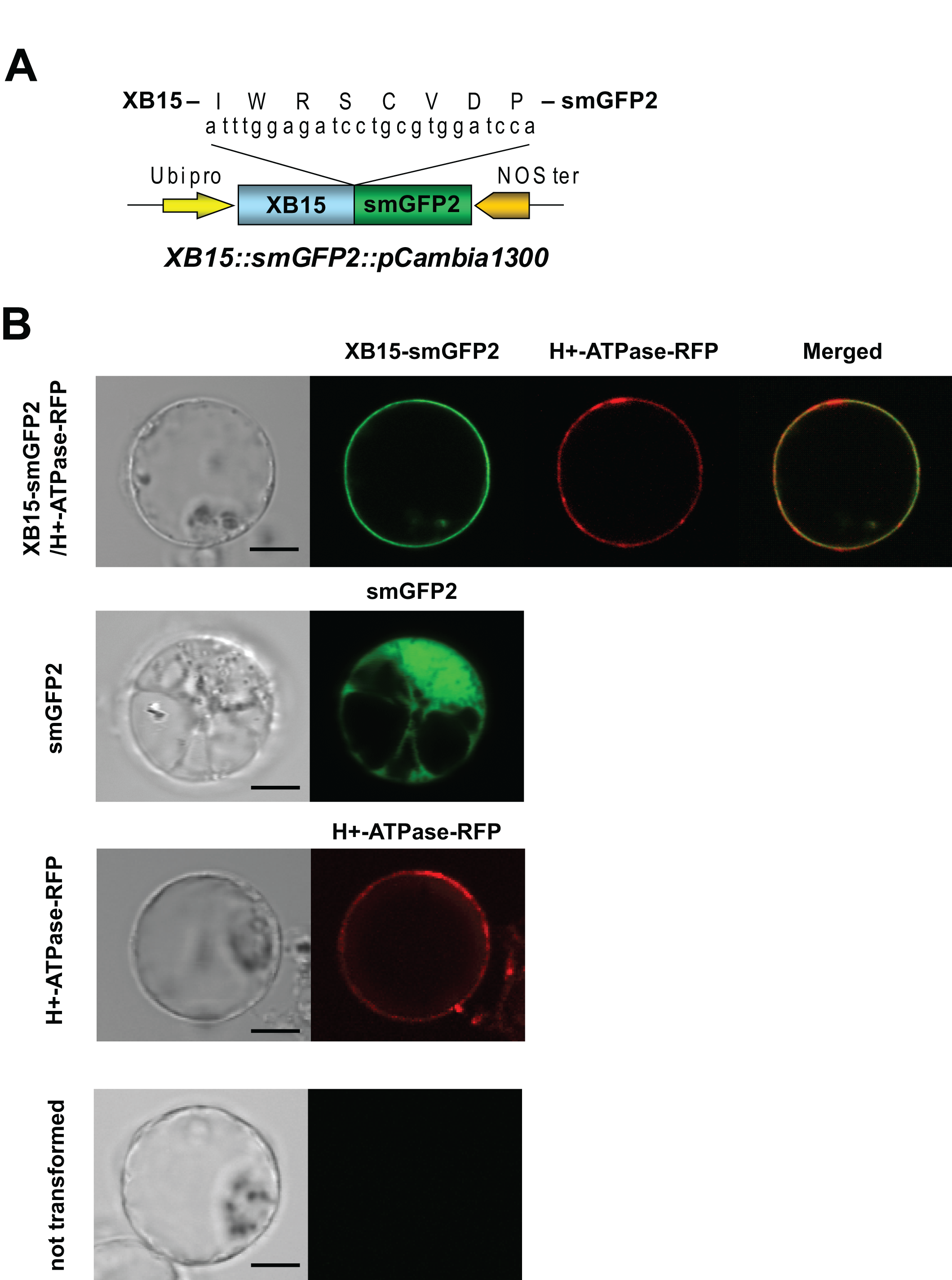

Supplement: Figure S4 — (A) Construction of Xb15-smGFP2 fusion gene. The PCR-amplified product without termination codon was fused in-frame to the coding region of smGFP2 [63,64]. Ubi pro, ubiquitin promoter; NOS ter, nopaline synthase terminator. (B) The Xb15-smGFP2 fusion, H+-ATPase-dsRed fusion, and control smGFP2 constructs were introduced into rice protoplast cells by PEG-mediated transformation [63]. H+-ATPase-dsRed was used as a marker for plasma membrane protein. Expressions of the introduced genes were observed 16 h after transformation. Images were collected with an Olympus FV1000 confocal microscope. The images were coded green (for smGFP2) or red (for dsRed). Scale bar, 10 μm. (4.98 MB TIF) [file pbio.0060231.sg004.tif]

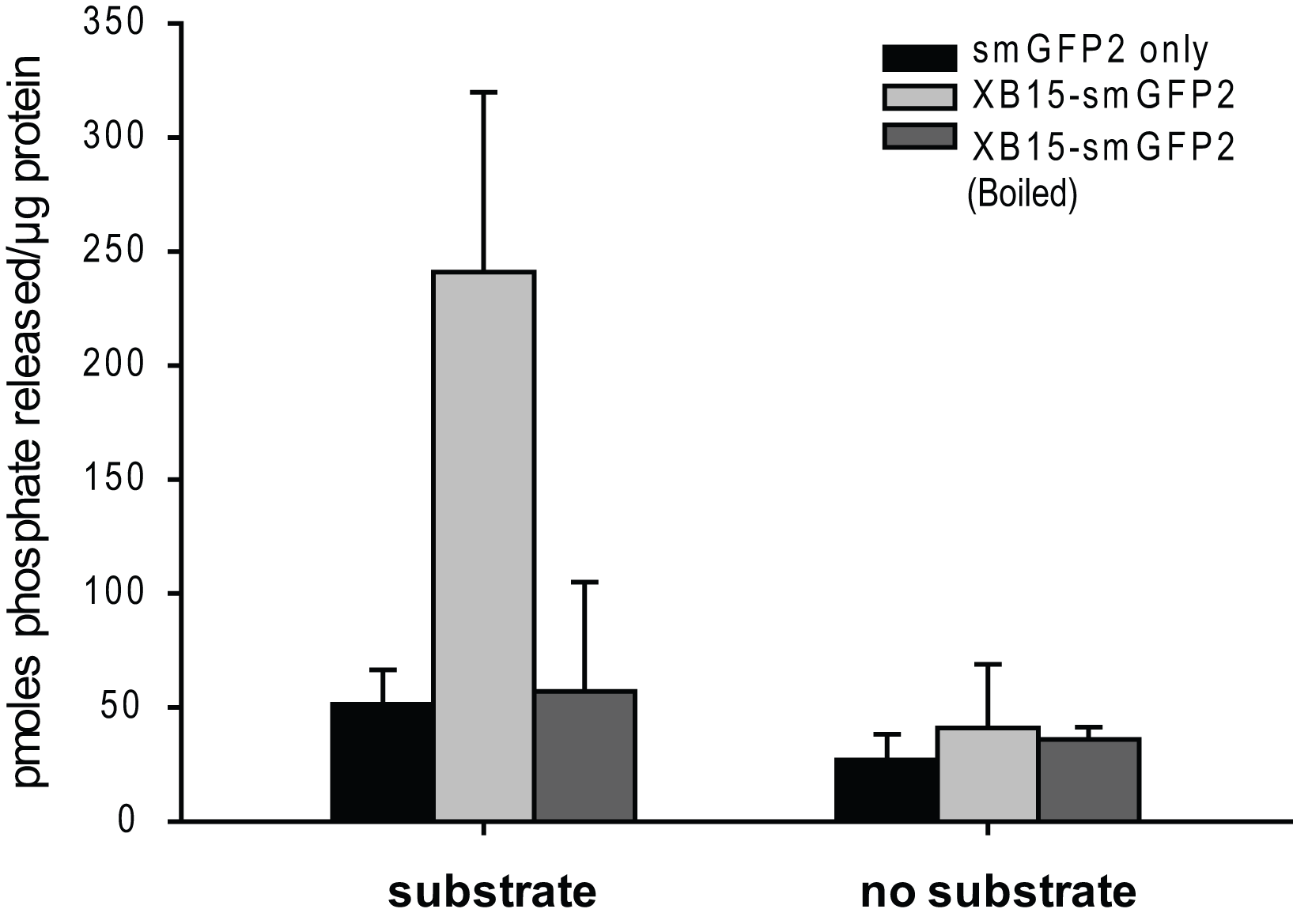

Supplement: Figure S5 — XB15-smGFP2 fusion protein and smGFP2 alone were purified from protoplasts transformed with each construct. Approximately 1 μg of each protein was incubated with 200 μM substrate in PP2C buffer for 60 min. For the boiled XB15-smGFP2, one μg of XB15-smGFP2 was boiled in water for 20 min. The data are average value of three experiments. Error bars represent standard deviations. (410 KB TIF) [file pbio.0060231.sg005.tif]
